# Plant G proteins: Versatile signaling hubs for synergistic improvement of agronomic traits

**DOI:** 10.1111/jipb.70351

**Published:** 2026-07-20

**Authors:** Haoran Li, Zhilong Zhang, Fangyuan Liu, Haonan Wang, Zebiao He, Sanyuan Tang, Xiaojing Dong, Peng Xie

**Affiliations:** ^1^ Guangdong Provincial Key Laboratory of Plant Stress Biology, School of Agriculture and Biotechnology Shenzhen Campus of Sun Yat‐sen University, Sun Yat‐sen University Shenzhen 518107 China; ^2^ Institute of Genetics and Developmental Biology Chinese Academy of Sciences Beijing 100101 China; ^3^ Yazhouwan National Laboratory Sanya 572024 China

**Keywords:** G proteins, regulatory network, stress response, trade‐offs, yield

## Abstract

Prioritizing defense responses often restricts growth and yield, forming a growth–defense conflict that limits crop productivity and threatens global food security. Traditionally attributed to resource allocation constraints, this trade‐off is governed by sophisticated signaling regulatory networks. Plant G proteins act as pivotal molecular switches that initiate cellular signal transduction, and they extensively integrate plant growth, development, and stress adaptation. Recent studies have uncovered their multifaceted coordination roles in regulating crop yield and stress resistance, and elite alleles of G proteins have been identified and applied in molecular design breeding. However, the pleiotropy of most G proteins hinders the concerted control of yield and stress resilience, leading to trait divergence, which represents a key bottleneck for breeding high‐yield and stress‐tolerant crops. Here, we summarize the regulatory networks of G protein subunits in yield and quality traits and stress responses, dissect the mechanisms underlying G protein‐mediated growth‐resistance decoupling, and propose precision strategies to simultaneously enhance these agronomic traits. This review provides theoretical and practical guidance for developing high‐performance crop varieties.

## Graphical abstract

This review highlights the roles of G‐protein signaling in crop yield and quality, as well as abiotic and biotic stress responses. It also discusses the feasibility of breaking the trade‐off between plant growth and stress tolerance via dissecting G‐protein regulatory pathways, gene editing, mining natural elite variants, and AI‐assisted breeding.

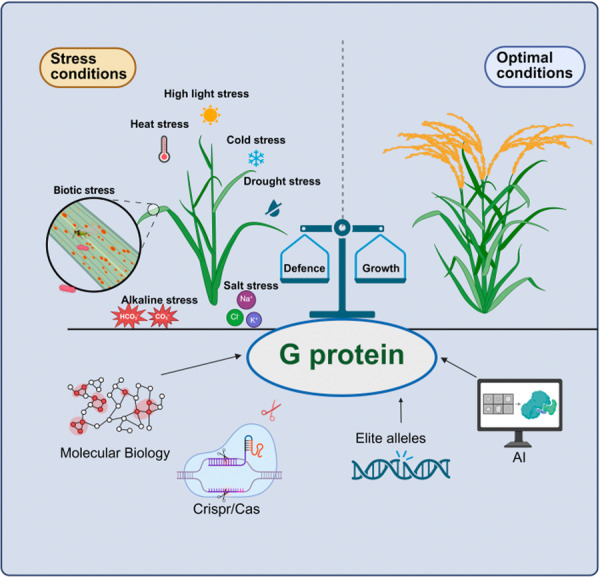

## INTRODUCTION

Ensuring global food security amid climate change and a growing population has become one of the most pressing challenges for modern agriculture. Specifically, climate change is projected to reduce maize, wheat, and soybean yields by 27.8%–35.6% by the end of the 21st century (Hultgren et al., 2025), while pests and diseases cause global yield losses of 30.0% for rice, 22.5% for maize, and 21.5% for wheat (Savary et al., 2019), and extreme drought already causes median yield losses of 10.1% in rainfed croplands (Tuninetti and Davis, 2026). Crop breeding aims to synergistically improve yield and stress resistance, but these two core agronomic traits are often mutually exclusive due to inherent growth–defense trade‐offs (Gao et al., 2024; 2024). When confronting abiotic stresses (e.g., drought, salinity, and extreme temperatures) or biotic threats (e.g., pathogens and pests), plants reallocate resources from growth and reproduction to defense‐related metabolism, often leading to significant yield penalties (Zhang et al., 2020). Breaking this trade‐off and achieving balanced improvement of yield and stress resistance have thus become key scientific bottlenecks in crop genetic improvement.

To cope with environmental fluctuations and coordinate growth–defense balance, plants have evolved sophisticated signaling networks, among which G protein signaling pathways stand out as conserved and versatile signal transduction hubs (Guo et al., 2025a). G proteins were first identified in mammals in 1980, defined as GTP‐binding proteins that initiate intracellular signaling cascades (Northup et al., 1980). Consisting of α, β, and γ subunits forming heterotrimeric complexes, G proteins are ubiquitous in eukaryotes, but plant G protein systems show unique structural and regulatory features. Unlike humans, who harbor 23 Gα, six Gβ, and 12 Gγ subunits (Milligan and Kostenis, 2006), most plants have a streamlined repertoire: Arabidopsis has one canonical Gα (GPA1), three extra‐large Gαs (XLG1‐3), one Gβ (AGB1), and three Gγ subunits (Temple and Jones, 2007); rice possesses one Gα (RGA1), one Gβ (RGB1), and five Gγ subunits (RGG1, RGG2, GS3, DEP1, GGC2) (Sun et al., 2018); and maize contains one Gα (CT2), one Gβ (MGB1), and six Gγ subunits (Wang et al., 2019a; Sun et al., 2026).

Another distinct feature of plant G protein signaling is the absence of canonical G protein‐coupled receptors (GPCRs). Instead, their functions are substituted by analogous proteins such as the maize CLAVATA receptor‐like protein (Bommert et al., 2013) and soybean nodulation factor receptors (Choudhury and Pandey, 2013). This deficit is further compensated by the intrinsic GDP–GTP exchange activity of plant Gα subunits (Urano et al., 2012), while Regulator of G Signaling (RGS) proteins fine‐tune G protein activity via GTPase acceleration (Ma et al., 2024). Phosphorylation of RGS and Gα subunits also plays a pivotal role in reactivating G protein signaling during stress responses (Liang et al., 2018; Jia et al., 2025), highlighting the complexity and specificity of plant G protein regulation.

Cumulative evidence has demonstrated that G proteins play dual and central roles in regulating both crop growth/development and stress resistance (Guo et al., 2025a). Crop yield, a complex trait determined by grain size, grain weight, panicle number, and grain number per panicle (Sakamoto and Matsuoka, 2008; Xing and Zhang, 2010), is tightly modulated by G protein subunits (Ma et al., 2024; Yaseen et al., 2025; Liang et al., 2025a). Meanwhile, G proteins are indispensable for plant responses to various stresses, including drought, salinity, extreme temperatures, and pathogens, by mediating stomatal dynamics, ROS homeostasis, hormone signaling, and immune activation (Ferrero‐Serrano and Assmann, 2016; Liu et al., 2025a). This unique ability to integrate endogenous developmental signals and exogenous environmental cues makes G proteins ideal targets for breaking the growth–defense trade‐off.

Against this backdrop, this review synthesizes the multifaceted roles of G proteins in coordinating yield and stress resistance, integrates the conserved and species‐specific regulatory networks underlying G protein‐orchestrated trait trade‐offs, and highlights emerging strategies for breaking this trade‐off. Finally, we discuss current challenges and future perspectives in exploiting G protein signaling for crop breeding, aiming to provide a comprehensive theoretical framework and practical targets for developing high‐yield, stress‐tolerant crop varieties.

## YIELD‐RELATED TRAITS

**Figure 1:**
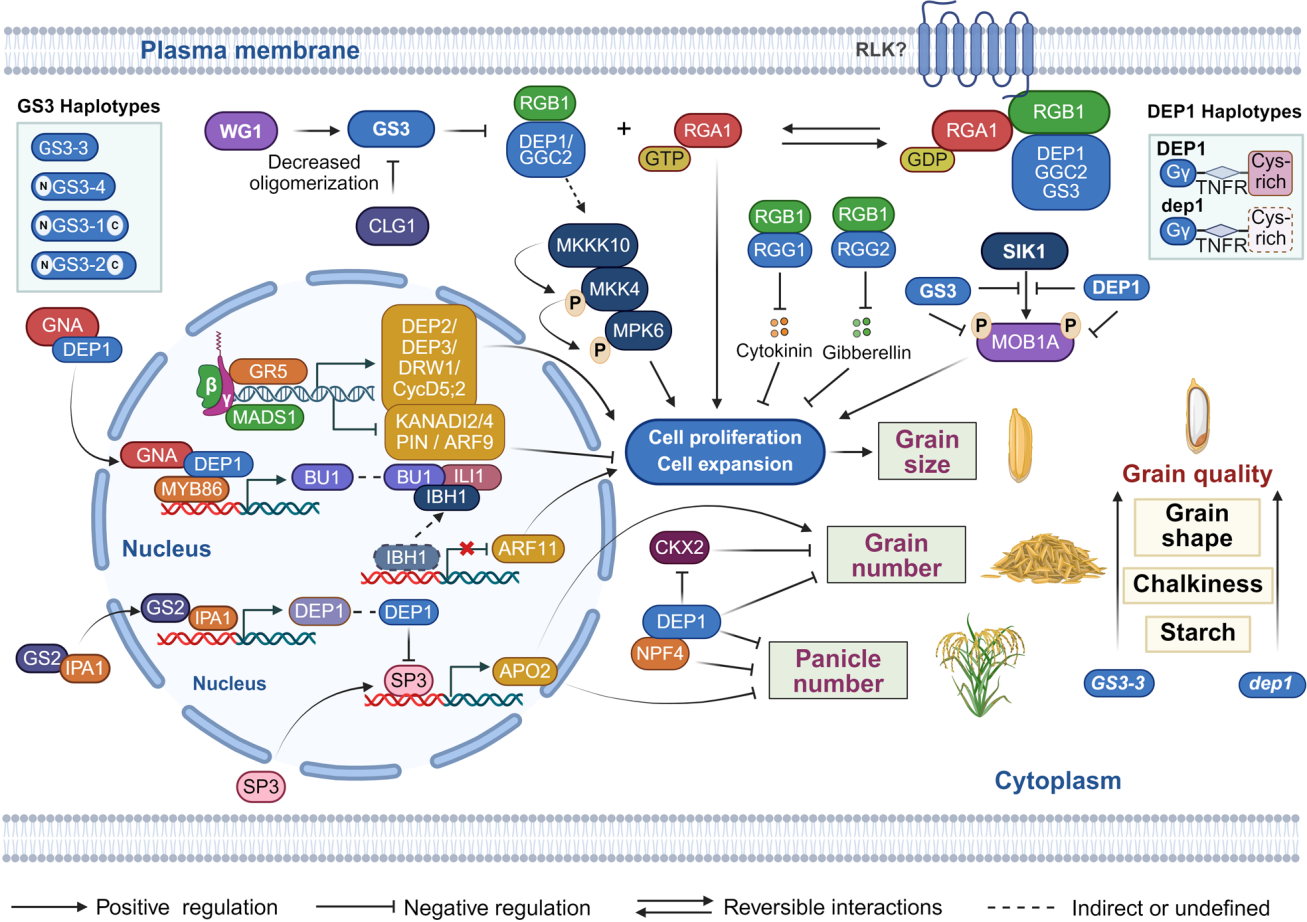
Regulation of crop yield and quality traits by G protein signaling components in rice Heterotrimeric G protein subunits (Gα, Gβ, and Gγ) regulate yield‐related traits including grain size, grain number per panicle, and panicle number through multiple mechanisms, as follows: (1) Modulation of phytohormone signaling pathways, including cytokinin, gibberellin, brassinosteroid, and other related phytohormones; (2) competitive binding of allelic variants of different Gγ subunits to the core Gα/Gβ complex; (3) synergistic action with downstream transcription factors (e.g., MADS‐box proteins, GR5) and protein kinases, which precisely modulate the patterns of cell division and expansion during grain development; and (4) the molecular network underlying the regulation of grain number and panicle number by the Gγ subunit *DEP1*. Grain quality traits are mainly modulated by *GS3* and *DEP1* through the regulation of grain shape, starch biosynthesis, and chalkiness formation. *GS3‐1* to *GS3‐4* represent four haplotypes of the rice *GS3* gene.

Crop yield is jointly determined by grain size and grain number per panicle (including tiller number and panicle development), both of which are core yield‐related traits and are precisely regulated by genetic networks integrating cell proliferation, cell expansion, and metabolic reprogramming (Ren et al., 2023; Yaseen et al., 2025). As conserved signaling hubs, heterotrimeric G protein subunits play species‐specific yet functionally interconnected roles in regulating grain size and grain number, with their regulatory polarity shaped by subunit interactions, structural variations, and crosstalk with hormonal and redox signaling. This chapter systematically summarizes the molecular mechanisms underlying G protein‐mediated grain size and grain number regulation, focusing on subunit‐specific functions, interspecies divergence, and downstream signaling integration (Figure 1; Table 1).

**Table 1:** G protein‐regulated genes associated with agronomic traits in crops

Species	G‐protein subunit(s)	Key interactors	Functions	References
Rice	XLGs (XLG1, XLG3)	OsCERK1 OsRLCK	*Osxlg1* mutants showed increased plant height, panicle length, and 1,000‐grain weight. *Osxlg3* mutants displayed reduced plant height, panicle length, and 1,000‐grain weight.	Zhao et al. (2022)
	Gα (RGA1)	RGB1 DEP1 GS3 bZIP47 COLD6	RGA1 positively regulates grain length and grain size, shade avoidance, and chalkiness degree. RGA1 negatively regulates drought tolerance, salt tolerance, and rice appearance quality.	Fujisawa et al. (1999), Ferrero‐Serrano et al. (2018), Oki et al. (2005), Jangam et al. (2016)
	Gβ (RGB1)	DEP1 GGC1 OsNF‐YB1	RGB1 positively regulates ABA response, drought adaptation, salt response, cell proliferation, seed fertility, grain size, and starch accumulation. RGB1 functions synergistically with *RGA1* and three Gγ subunits to control grain length; *RGB1* knockout causes dwarf phenotype and increased sterile seeds.	Sun et al. (2018), Liu et al. (2018b), Biswas et al. (2019), Zhang et al. (2015)
	Gγ (RGG1, RGG2)	RGA1 RGB1 DGS1	RGG1 and RGG2 negatively regulate grain size and positively regulate ABA response and salt response.	Tao et al. (2020), Swain et al. (2017), Yadav et al. (2012)
	Gγ(GS3/TT2)	RGA1 RGB1 OsSCT1 OsWR2 WG1 DGK7 MADS1 SIK1 OsPIP2;1/2;2	GS3/TT2 negatively regulates grain length, grain size, grain weight, stigma exsertion, and heat and alkaline tolerance, and suppresses brown planthopper resistance; meanwhile, loss‐of‐function allele *gs3‐3* positively improves rice appearance quality.	Kan et al. (2025), Fang et al. (2025a), Shen et al. (2023), Zhang et al. (2023), Huang et al. (2022)
	Gγ(DEP1)	RGA1 RGB1 OsMADS1 OsGNA OsLPA1 OsSP3 OsSIK1	DEP1 positively regulates grain number, panicle density, yield, BR signaling, and sheath blight resistance, negatively regulates plant height, panicle length, tiller angle, and drought response, and elite DEP1 variants *dep1* optimize the trade‐off between yield and grain quality.	Li et al. (2025a), Zhang et al. (2025b), Zhou et al. (2025a), Liu et al. (2025b), Sun et al. (2014)
	Gγ(GGC2)	RGA1 RGB1	GGC2 positively regulates grain length and plant height.	Sun et al. (2018)
Maize	XLGs (XLG1, XLG3a, XLG3b)	ZmGβγ ZmPR1 ZmPR5	Single *xlg* mutants show normal developmental phenotypes. All double *xlg* mutant combinations cause dwarfism. *Zmxlg1;3a;b* triple mutants were lethal at the seedling stage and high expression of *PR1* and *PR5* (*Pathogenesis‐Related*) genes.	Wu et al. (2018), Cantos et al. (2023)
	Gα (CT2)	FEA2 MGB1	Expression of a constitutively active *CT2* resulted in higher spikelet density and kernel row number. The hundred‐kernel weight of *ct2* decreased drastically.	Wu et al. (2018), Shi et al. (2025)
	Gβ (MGB1)	CT2 XLGs	*MGB1*‐knockout lines show seedling lethality caused by autoimmune activation. Natural variation in *MGB1* influences meristem size and kernel row number.	Wu et al. (2020)
	Gγ (MGG1, MGG3, MGG4)	CT2 MGB1	MGG1 negatively regulates kernel number. MGG3 influences maize kernel development. MGG4 positively regulates kernel size.	Li et al. (2025b), (2010), Zhao et al. (2025a)
Wheat	Gα (TaGα)	TaMLO1 TaGSTU51	*TaGα‐aabbdd* mutants reduces spike length, grain length, and thousand‐grain weight.	Zhang et al. (2025a), Li et al. (2026)
	Gβ (TaGB1‐B)	NA	TaGB1‐B *e*nhances drought and salt resistance in wheat.	Xiong et al. (2023)
	Gγ (DEP1)	NA	*DEP1*‐knockout lines show decreased grain size and thousand‐grain weight.	Li et al. (2022a)
	Gγ (AT1/GS3)	PIP2	The N‐terminal Gγ domain of AT1/GC1 negatively regulates alkaline tolerance.	Zhang et al. (2023)
Tomato	Gβ (SlGB1)	SlBHLH79 SlRGS1	SlGB1 governs tomato shoot apex growth and development and *SlGB1* mutants display constitutive autoimmune responses.	Wang et al. (2025b), Ninh et al. (2021)
	Gγ (SlGGB1, SlGGC1)	SlGB1	Silencing of *SlGGB1* results in smaller seeds, higher number of lateral roots, and pointy fruits. The *Slggc1* mutant shows increased yield and improved fruit quality.	Subramaniam et al. (2016), Liang et al. (2025b)
Mustard	Gα (BjuGα1)	NA	BjuGα1 positively regulates vegetative and reproductive growth traits, while constitutively active Gα enhances these growth‐related characteristics.	Kumar and Bisht (2020)
Cucumber	Gα (CsGPA1)	CsCLV1	The *Csgpa1* mutant displays increased floral organ numbers and shorter and wider fruits.	Han et al. (2024)
Sorghum	Gγ(AT1/GC1)	SbPIP2;1 SbpPLAII‐1	The N‐terminal Gγ domain of AT1/GC1 positively regulates glume coverage and negatively regulates alkaline tolerance.	Zou et al. (2020), Xie et al. (2022), Zhang et al. (2023)
Millet	Gγ (SiGC1)	NA	The Gγ domain of SiAT1 negatively regulates glume length, hard‐threshing ability, and alkaline tolerance.	Xie et al. (2022)

### Gα subunits: Conserved positive regulation across crops

Gα subunits universally act as positive regulators of yield‐associated traits in major crops, with their GTPase activity and interaction with downstream effectors governing cell division or expansion dynamics. In rice, RGA1 modulates grain size and panicle development by mediating GA signaling to regulate cell division. Loss‐of‐function *rga1* (*d1*) mutants show reduced grain length and grain weight (Ueguchi‐Tanaka et al., 2000; Yang et al., 2024). This mechanism is conserved in wheat: the triple mutant *TaGα‐aabbdd* shows reduced grain and spike length, and thousand‐kernel weight (TKW) (Zhang et al., 2025a). Maize Gα (CT2) further expands the regulatory network by integrating the CLAVATA pathway: CT2 physically interacts with the LRR receptor‐like protein FEA2 and transmits stem cell restriction signals to control the sizes of shoot apical meristem (SAM) and inflorescence meristem (IM), thereby shaping maize yield architecture (Bommert et al., 2013). *ct2* mutants display significantly smaller kernels (Shi et al., 2025), while constitutively active *CT2* increases kernel density and kernel row number (Wu et al., 2018). Even in dicots like mustard (*Brassica juncea*), overexpression of a constitutively active *BjuGα1* enhances seed weight by 7%–25% (Kumar and Bisht, 2020), highlighting the conserved role of Gα in linking hormone signaling to grain size.

### Gβ subunits: Species‐specific regulation constrained by metabolic pathways

The Gβ subunit regulates yield traits bidirectionally and in a species‐specific manner, closely relying on its interactions with distinct Gγ subunits. In rice, RGB1 is essential for grain length control. It positively regulates grain elongation via binding to DEP1/GGC2, whereas GS3 interaction antagonizes such effects and reduces grain size. Both overexpression and silencing of *RGB1* alone suppress grain development (Sun et al., 2018; Liu et al., 2018b). Notably, Gβ knockout causes seedling lethality in monocots (rice, maize) and tomato, resulting from constitutive autoimmune activation and disrupted phenolamide homeostasis (Wu et al., 2020; Wang et al., 2025b). In tomato, SlGB1 modulates phenolamide biosynthesis through the bHLH79/MYB10–THT5/8 module. Viable biallelic *SlGB1* mutant lines (*Slgb1_d6i1*) produce smaller fruits and seeds, thereby markedly reducing crop yield. In contrast, *Arabidopsis agb1* mutants lacking functional Gβ remain viable with mild developmental defects, as Arabidopsis lacks the THT (tyramine hydroxycinnamoyl transferase)‐associated phenolamides metabolic pathway (Ullah et al., 2002; Ninh et al., 2021). Such divergence reflects the dual roles of Gβ in balancing plant growth and immunity. In THT‐containing species, Gβ suppresses autoimmune signaling and phenolamide synthesis, and its loss triggers aberrant immunity and metabolic cytotoxicity.

### Gγ subunits: Structural‐dependent functional diversification

Heterotrimeric Gγ subunits show the highest functional diversity among rice yield regulators, with their regulatory polarity governed by a highly conserved N‐terminal Gγ‐like domain and a plant‐specific variable C‐terminal tail. Rice encodes five distinct Gγ subunits: The tailless (lacking C‐terminal cysteine‐rich domains) Type‐A subunit RGG1 and Type‐B subunit RGG2 function as negative regulators of grain size and weight. Specifically, RGG1 inhibits cell division by reducing cytokinin (CK) levels (Tao et al., 2020), whereas RGG2 suppresses cell expansion by modulating gibberellin (GA) biosynthesis and signaling pathways (Miao et al., 2019). In contrast, the three atypical Gγ subunits—GS3, DEP1, and GGC2—function through tail‐mediated protein stability. The positive regulators DEP1 and GGC2 possess long C‐terminal tails containing multiple tandem repeats that promote cell proliferation in grain hulls and panicles. Conversely, the short‐tailed GS3 acts as a negative regulator by competitively binding to Gβ against DEP1 and GGC2, thereby indirectly inhibiting their functions and shortening grain length (Sun et al., 2018, 2025). The positive regulatory role of DEP1 orthologs in grain size and weight has been further validated in sorghum (Tao et al., 2021), wheat (Li et al., 2022a), and maize (Zhao et al., 2025a), suggesting that long‐tailed DEP1‐like Gγ subunits are evolutionarily conserved across monocot crops.

The functional importance of these domains is underscored by the fact that loss of function in the conserved N‐terminal Gγ‐like domain shared by all five subunits leads to increased grain length, while overexpression of truncated Gγ variants lacking any C‐terminal tail results in a drastic reduction in grain size. This suggests that the conserved Gγ‐like domain serves as a core negative regulatory module. The variation in grain size associated with the C‐terminal tail is partially attributed to alterations in protein stability, as the tail is a target for ubiquitination by the RING‐type E3 ligase CLG1 (Yang et al., 2021). For instance, *GS3‐3*, a rare natural variant of *GS3* that lacks the C‐terminal tail, escapes CLG1‐mediated degradation, resulting in a long‐grain phenotype. This variant has become one of the most effective elite alleles in rice breeding for improving grain length and weight (Mao et al., 2010).

Beyond regulating grain size, the rice Gγ subunit DEP1 plays a pivotal role in determining panicle number (governed by tiller outgrowth) and grain number per panicle, which is influenced by panicle branching and spikelet fertility. Since its identification as the major quantitative trait locus (QTL) for the erect panicle phenotype in rice (Huang et al., 2009), recent studies have uncovered new layers of DEP1‐mediated crosstalk between brassinosteroid (BR) and CK signaling (Li et al., 2025a; Zhang et al., 2025b).

Given the inherent self‐activating nature of plant G‐protein signaling pathways, it is hypothesized that *GS3* acts as a “brake pad” to fine‐tune signaling by preventing the overactivation of *DEP1* and *GGC2*. This Gγ tail‐driven regulatory mechanism is conserved in other gramineous crops. For example, MGG3 in maize and GC1 in sorghum also negatively regulate grain size and yield (Li et al., 2010; Zou et al., 2020). Furthermore, this G‐protein machinery underpins floral organ development, regulating glume coverage and grain threshability in sorghum and millet (Xie et al., 2022; Xie et al., 2023). However, loss‐of‐function elite alleles that confer similar yield advantages have not yet been extensively selected or preserved through the process of artificial evolution in these crops.

### G‐protein regulatory signaling network: Integration of transcriptional, redox, and hormonal signals

G‐protein‐mediated yield regulation converges upon a sophisticated signaling network that integrates transcriptional control, kinase phosphorylation cascades, redox homeostasis, and crosstalk among multiple phytohormones, including BR, CK, and auxins.

At the transcriptional level, Gγ subunits form diverse regulatory modules to drive development. Specifically, GS3 and DEP1 interact directly with the downstream transcription factor OsMADS1, acting as co‐factors to enhance its activity and co‐activate target genes that determine grain size and weight (Liu et al., 2018b). This pathway is further diversified by natural variations, such as a rare spontaneous mutation in *OsMADS1* that induces alternative splicing to relieve the inhibition of cell proliferation, thereby increasing grain length (Wang et al., 2019b). Similarly, the AP2‐family transcription factor GR5 interacts differentially with distinct functional types of Gγ subunits to form a Gγ–GR5 module, which modulates grain cell proliferation and longitudinal expansion (Wang et al., 2024a). The expression of *DEP1* itself is fine‐tuned by an upstream activation complex composed of GS2 (a GRF factor) and IPA1 (an SPL factor), which triggers DEP1 to promote panicle branching and increase grain number (Wang et al., 2025a).

Beyond transcription, G‐protein signaling is heavily influenced by physical protein interactions and post‐translational modifications. DEP1 and GS3 physically interact with the Hippo pathway component OsSIK1, affecting the OsSIK1–OsMOB1A association and the phosphorylation of OsMOB1A to balance grain dimensions (Zhou et al., 2025b). Additionally, DEP1 interacts with the nitrate transporter OsNPF4 to couple nutrient utilization with grain number regulation (Chen et al., 2025a). The cysteine‐rich C‐terminus of DEP1 interacts with SP3, regulating its shuttling between the plasma membrane and the nucleus, activating *APO2* expression, and promoting spike branching (Liu et al., 2025b). On the post‐translational front, the glutathione peroxidase WG1 provides an additional layer of post‐translational control by modulating the oligomerization state of GS3 in a redox‐dependent manner (Liu et al., 2025a).

Hormonal crosstalk represents another critical dimension of this network, with *DEP1* serving as a central hub. As BR levels increase, the GRAS‐family protein GNA facilitates the nuclear localization of DEP1. Once in the nucleus, a DEP1–GNA–OsMYB86 complex forms to activate the transcription of *BU1*, a positive regulator of the BR response. BR further promotes the nuclear import of BU1 through the atypical bHLH factor ILI1, which subsequently competes with IBH1 to interact with ILI1. This synergy relieves the repression of *OsARF11* by the *BU1–ILI1–IBH1* module, ultimately activating BR and auxin responses to influence panicle traits (Li et al., 2025a). Simultaneously, the increased nuclear DEP1–GNA complex binds to the promoter of the cytokinin oxidase *OsCKX2/Gn1a*, a key negative regulator, to inhibit its transcription. This inhibition results in the accumulation of CK in the inflorescence meristem, thereby increasing reproductive organ number and grain count per panicle (Zhang et al., 2025b). This mechanism explains the success of the *dep1* gain‐of‐function allele, which lacks a C‐terminal tail and is widely used in breeding to increase overall grain yield per plant by 40.9% without compromising grain size (Huang et al., 2009).

Collectively, G‐protein‐mediated regulation of yield traits operates through three core mechanisms: (i) The competitive binding between Gγ variants and the Gβ complex, (ii) synergistic interactions with transcription factors and kinases to modulate downstream targets, and (iii) the dynamic regulation of Gγ subunits through post‐translational modifications such as ubiquitination and redox‐state changes.

## G PROTEIN‐MEDIATED REGULATION OF CROP QUALITY TRAITS

The G‐protein β subunit RGB1 can indirectly regulate the expression of *OsNF‐YB1* and *OsYUC11* genes through an as‐yet‐unidentified mechanism, thereby participating in endosperm starch accumulation and auxin biosynthesis to control grain shape (Zhang et al., 2021). Optimization of grain shape can effectively reduce chalkiness rate and chalkiness area, thereby significantly improving the appearance, taste, and other quality characteristics of rice. In addition, the rice *GS3* gene may have undergone artificial selection; after introducing the *gs3* allele into the rice variety “Nanyangzhan”, the resulting plants produced extra‐long grains, with a significant increase in grain width, grain thickness, and thousand‐kernel weight (TGW) (Huang et al., 2022). In another study, pyramiding the *gw8* and *gs3* alleles into the short and wide‐grain rice variety “HJX74” successfully yielded the elite variety “Huabiao1”, which has elongated grains, significantly improved quality, and no impact on yield (Wang et al., 2012). The transcription factor OsMADS1 can directly interact with the G‐protein γ subunits DEP1 and GS3 to jointly regulate the expression of downstream target genes. Pyramiding the nature allele *OsMADS1* and the high‐yield gene *depl* into conventional rice breeding can significantly improve rice quality (Liu et al., 2018b).

Several elite rice varieties have been obtained through pyramiding of beneficial alleles related to grain traits. For example, “Yunguang 8” carries two beneficial alleles, *gw8* ^*Basmati*^ and *OsLG3* ^*SLG*^, and has both high yield potential and excellent appearance quality (Yu et al., 2018a). By pyramiding the semi‐dominant *gs3* allele and the *GW7* ^*TFA*^ allele derived from tropical japonica varieties, two new hybrid indica rice varieties, “Taifengyou 55” and “Taifengyou 208”, were developed, and both varieties showed significant improvements in yield and quality (Wang et al., 2015).

Varieties of the Jiahe series, such as Jiahe 212 and Jiahe 218, have excellent appearance quality due to the pyramiding of *dep1* and *gs3* genes. Notably, the new temperate long‐grain hybrid japonica rice variety “Jiahe 218” was bred by pyramiding three beneficial alleles: *gs3*, *GW7* ^*TFA*^, and *dep1‐2*. This variety not only has slender grains and low chalkiness but also effectively solves the trade‐off between grain length and head rice rate (Huang and Qian, 2017). Another variety, “Jiafengyou 2”, carries the *gs3* and *dep1* genes from sterile lines and the *IPA1‐2* gene from restorer lines, resulting in a significant increase in yield (Huang and Qian, 2017). “Zhongke 804” is a high‐yielding and highly disease‐resistant rice variety, bred by pyramiding the *gs3* gene and the *Pi5* gene, achieving synergistic improvement of yield and disease resistance. By leveraging the indica–japonica hybrid advantage and ideal plant architecture genes, the japonica hybrid rice variety “Jiaheyou 7245” was bred through pyramiding the *GL7* and *IPA1* genes; at the same time, the new long‐grain japonica variety “Zhongjia 8”, which has excellent grain shape, quality, plant architecture, and yield, was developed by pyramiding the *gs3* and *dep1* genes (Zeng et al., 2017). All these varieties demonstrate the great potential of G protein subunits in improving crop quality.

## G PROTEIN‐MEDIATED REGULATION OF CROP STRESS RESISTANCE

**Figure 2:**
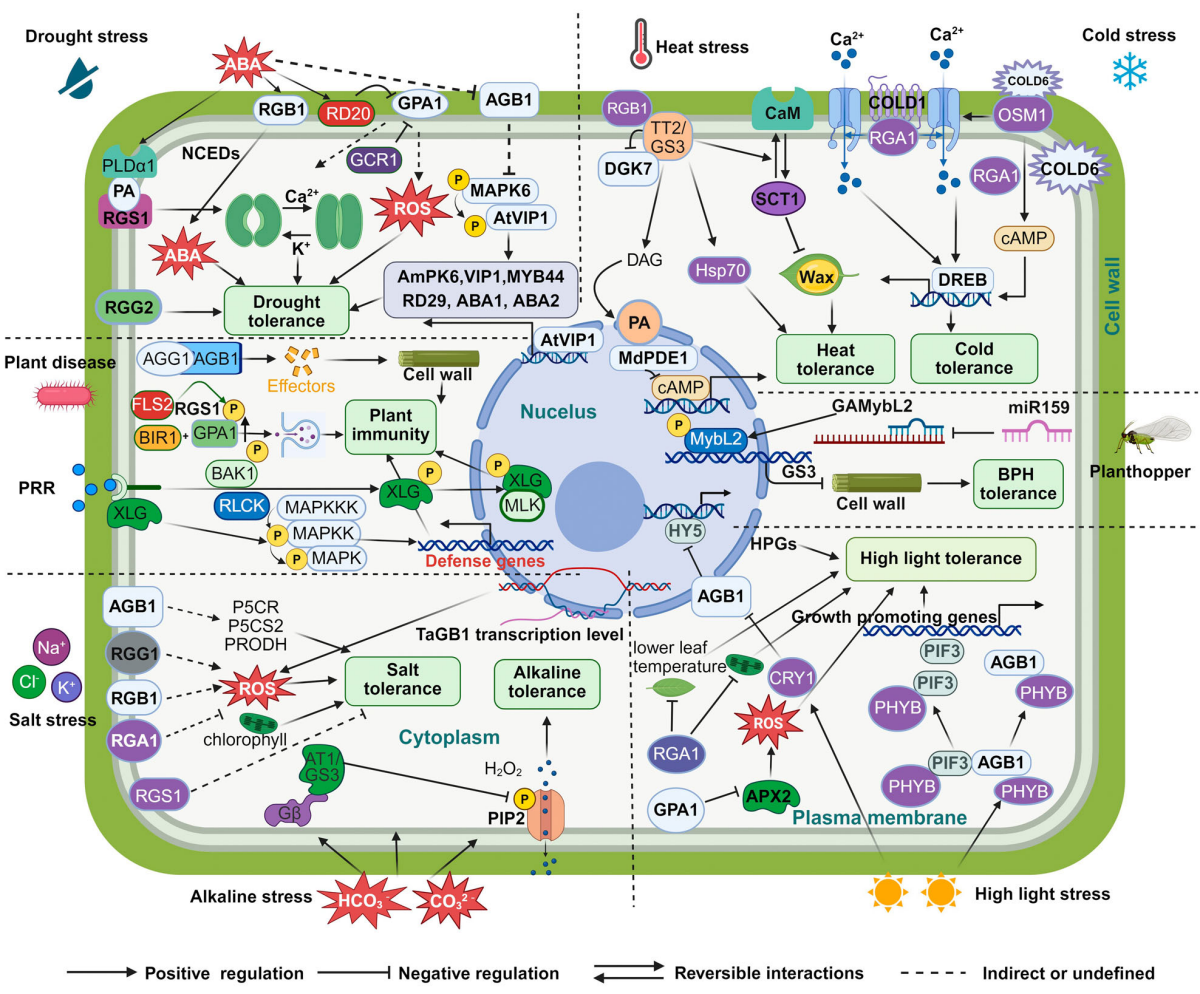
Regulatory network of G proteins in plant stress responses G protein components sense stress signals (drought, temperature, salinity, and light) and transduce them to downstream effector molecules, thereby initiating adaptive physiological responses in plants. (1) Drought stress: Water stress induces ABA accumulation, which activates G proteins (GPA1 and AGB1) via the PLDα1–PA–RGS1 and MAPK pathway. This further regulates guard cell ion channels (K^+^ and Ca^2+^) and downstream stress‐responsive genes (e.g., *RD29A* and *ABI1*), enhancing plant drought tolerance. (2) Heat/cold stress: G protein subunits (AGB1, RGA1, and TT2) participate in heat/cold tolerance signaling by regulating ion transport, molecular chaperones (Hsp70), and transcription factors (AP2‐type transcription factors) and the cAMP pathway. (3) Saline–alkali stress: G proteins modulate saline–alkali ion accumulation and saline‐alkali tolerance by modulating ROS metabolism, ion transporters (PIP2). (4) High light stress: G proteins (AGB1, GPA1, and RGA1) balance growth and stress tolerance under high light by repressing growth‐promoting genes, high‐light avoidance (*RGA1*), and scavenging reactive oxygen species (ROS). The arrows and blunt arrows represent positive and negative regulation, respectively. Double arrows represent reversible bindings. (5) Plant disease: *XLGs* initiate downstream defense responses via the MAPK cascade. Meanwhile, XLG interacts with MLK, translocates to the nucleus, and activates plant immunity. FLS2 perceives pathogen signals and BIK1 phosphorylates the RGS1 protein. This leads to the dissociation of RGS1 from the Gα subunit and FLS2, thereby activating the Gα subunit to promote immune responses. Furthermore, *AGB1* can regulate leaf mechanical strength through its interaction with AGG1, thereby influencing pathogen invasion. (6) Brown Planthopper: Non‐coding RNA and transcriptional regulation: *miR159* targets *GAMYBL2* and participates in GS3‐mediated regulation of cell wall synthesis, further synergistically enhancing plant resistance to Brown Planthopper (BPH) Resistance.

As sessile organisms, plants rely on G protein signaling to integrate abiotic and biotic stress cues into adaptive responses. G proteins modulate stress tolerance via conserved pathways (e.g., ROS homeostasis and hormone signaling) and species‐specific adaptations, directly contributing to the growth–defense trade‐off. Below, we synthesize G protein functions in key stress responses and their implications for crop improvement (Figure 2; Table 1).

### Abiotic stress resistance

Abiotic stresses including drought, salinity, extreme temperatures, and high light are the primary environmental factors restricting crop growth and yield formation worldwide. G proteins, as conserved and versatile signal hubs, can sense and transduce various abiotic stress signals, and regulate a series of adaptive physiological and biochemical responses in crops through multiple signaling pathways, thus endowing crops with tolerance to adverse environments.

#### Drought stress

Drought stress poses a severe threat to global crop yield and food security, and plants have evolved intricate signaling networks to cope with water deficit, among which G protein‐mediated signaling acts as a conserved and pivotal module. G proteins orchestrate multiple drought‐adaptive physiological processes, including stomatal dynamics, ROS homeostasis, and ABA signaling, with distinct regulatory patterns and subunit‐specific functions observed across plant species.

In Arabidopsis, the Gα subunit GPA1 serves as a central positive regulator of drought tolerance, mainly by mediating ABA‐induced stomatal closure; accordingly, gpa1 mutants display reduced ABA sensitivity, impaired ABA‐triggered stomatal closure, and elevated water loss rates, alongside defects in multiple ABA‐dependent processes such as K^+^ influx inhibition, anion channel activation, Ca^2+^ channel stimulation, and ROS production (Wang et al., 2001; Nilson and Assmann, 2010; Guo et al., 2025c). GPA1 also modulates stomatal density by governing epidermal cell development and stomatal formation, with *gpa1* mutants showing altered stomatal traits and transpiration efficiency. Mechanistically, GPA1 interacts with phospholipase Dα1 (PLDα1) to enhance its GTPase activity, while phosphatidic acid (PA) produced by PLDα1 binds and inhibits RGS1, a GTPase‐activating protein, forming a negative feedback loop that fine‐tunes Gα activity (Zhao and Wang, 2004; Roy Choudhury and Pandey, 2016). Further regulators of GPA1 include the calcium‐binding protein RD20/CLO3 and the putative G protein‐coupled receptor GCR1, both of which act as negative modulators in GPA1‐mediated ABA and drought signaling (Brunetti et al., 2021). The Gβ subunit AGB1 negatively regulates ABA signaling and drought tolerance via the *MAPK6–AtVIP1–MYB44* cascade. Upon stress, AGB1‐Gγ activates MAPK6, which phosphorylates AtVIP1 to modulate the expression of stress genes (*MYB44*, *RD29*, and *ABA1/2*). The *agb1* mutant shows enhanced ABA sensitivity and drought resistance, confirming this module as a key drought adaptation pathway (Xu et al., 2015).

In contrast, rice shows clear species‐specific G protein signaling: The *d1* mutant, which harbors a loss‐of‐function allele of the Gα gene *RGA1*, confers enhanced drought tolerance through an erect plant architecture that lowers leaf temperature and reduces the transpiration driving force, as well as delayed stomatal limitation to photosynthesis, representing a distinct regulatory strategy from Arabidopsis (Ferrero‐Serrano and Assmann, 2016). The Gγ subunit *RGG2* is transcriptionally and post‐transcriptionally induced by ABA and drought stress in a time‐ and intensity‐dependent manner, and acts synergistically with Gα and Gβ to upregulate stress‐responsive genes, reinforce ROS scavenging capacity, and coordinate stomatal behavior, thereby enhancing drought resilience (Miao et al., 2024). Another Gγ subunit DEP1 is regulated by RGB1, and acts as a negative regulator in ABA‐mediated drought stress responses (Zhang et al., 2015).

Heterologous expression assays in tobacco further support functional divergence among plant G protein subunits: Ectopic expression of maize *MaGα* compromises drought and salt tolerance, whereas overexpression of *MaGβ*, *MaGγ1*, *MaGγ2*, or wheat *TaGB1* enhances stress resistance (Liu et al., 2019; Xiong et al., 2023). Collectively, these findings illustrate both the conserved core roles and extensive species‐specific diversification of G protein signaling in plant drought adaptation, underscoring their potential value for genetic improvement of stress‐resistant crops.

#### Saline–alkali stress

Saline–alkali stress (high salt and high pH) is a major abiotic stress in agricultural production. It causes dual damage to plants mainly through osmotic stress, ion toxicity, disruption of root cell membrane structure, and inhibition of nutrient uptake, thereby posing a severe threat to global food security (Xiao et al., 2024). As a conserved signal regulatory module, G proteins participate in plant responses to saline–alkali stress by regulating key physiological processes such as ion homeostasis and reactive oxygen species (ROS) metabolism, and their α, β, and γ subunits serve differential regulatory functions across different species.

In *Arabidopsis thaliana*, Gα subunits negatively regulate salt tolerance, and loss‐of‐function mutants show significantly reduced salt‐induced cellular senescence and growth inhibition. This regulation is specifically achieved by alleviating cell cycle inhibition caused by osmotic stress and mediating leaf senescence and cell death induced by Na^+^ toxicity (Colaneri et al., 2014). The Gβ subunit AGB1 acts as a positive regulator of salt tolerance; high salt stress can significantly upregulate its transcript level, and the *AGB1* loss‐of‐function mutant (*agb1*) shows increased salt sensitivity, characterized by decreased chlorophyll content, proline accumulation, and peroxidase (POD) activity, as well as increased malondialdehyde (MDA) content and Na^+^/K^+^ ratio. The regulatory mechanism of AGB1 involves multiple pathways, including regulating the expression of genes related to osmotic adjustment (*P5CR*, *P5CS2*, and *PRODH*), oxidative stress (*POD* and *APX1*), ion homeostasis (*HKT1* and *NHX1*), and stress response (*ZAT12, CBF3*, etc.), participating in the SOS pathway to maintain ion homeostasis. The *agb1/fer* double mutant showed a phenotype analogous to that of the *sos1* mutant, a key mutant for the SOS pathway (Yu and Assmann, 2018). In the *agb1* single mutant, the expression of *CBL8*, a calcium signaling component essential for the SOS cascade, was markedly downregulated (Yadav et al., 2024). This change disrupted SOS signal transduction, impaired the plant's capacity to sustain ionic homeostasis, and ultimately reduced salt stress tolerance. In brief, AGB1 preserves the protein abundance of CBL8 to maintain intact SOS pathway activity, thereby enhancing plant adaptation to salt stress. AGB1 inhibits Na^+^ translocation from roots to shoots by regulating stomatal aperture and conductance, and cooperates with the receptor‐like kinase FERONIA (FER) in ROS production during salt stress responses (Ma et al., 2015a; Yu and Assmann, 2018). In addition, for the GTPase‐activating protein AtRGS1, a negative regulator of G protein signaling, one of its loss‐of‐function mutant (*rgs1‐2*) shows enhanced Na^+^ tolerance, consistent with the negative regulatory role of AtRGS1 in G protein signaling (Colaneri et al., 2014).

In staple food crops, the regulatory functions of G protein subunits also show species specificity: In *Oryza sativa* (rice), the Gα subunit RGA1 negatively regulates salt tolerance, and its loss‐of‐function mutants (*d1*, *sd58*) enhance salt tolerance by balancing photosynthesis and ROS accumulation, specifically reducing oxidative damage by regulating ROS‐mediated stomatal conductance (affecting photosynthetic efficiency) and antioxidant enzyme activity (maintaining ROS homeostasis) (Peng et al., 2019). The double mutant of atypical Gα subunits (*xlg1/xlg4*) also shows improved salt tolerance (Cantos et al., 2023). The Gβ subunit RGB1 positively regulates salt tolerance by reducing electrolyte leakage and MDA content, and its overexpression can upregulate the expression of stress‐responsive genes (Biswas et al., 2019). Overexpression of *RGG1* enhances salt tolerance by upregulating the expression and activity of antioxidant genes such as *CAT*, *APX*, and *GR*, thereby strengthening ROS scavenging capacity (Liu et al., 2022). In Zea mays (maize), Gα subunits also negatively regulate salt tolerance similar to that in Arabidopsis; their loss‐of‐function mutants show alleviated leaf senescence, chlorophyll degradation, electrolyte leakage, and growth inhibition under NaCl stress (Urano et al., 2014).

Compared with studies on salt stress, alkaline stress, characterized by high pH and toxic levels of carbonate/bicarbonate ions, has become a new research focus. Gγ subunit AT1 (a homolog of rice OsGS3 and maize ZmGS3) in *Sorghum bicolor* is a key negative regulator of alkaline tolerance. SbAT1 exerts its inhibitory effect by forming a complex with Gβ, which in turn suppresses the phosphorylation of aquaporin PIP2. This reduced phosphorylation impairs PIP2's function as a H_2_O_2_ exporter, leading to excessive accumulation of ROS in cells and ultimately increasing sorghum's sensitivity to alkaline stress (Zhang et al., 2023). Notably, genetic manipulation of *AT1*—either by knocking out its homologous genes or using natural non‐functional variants—significantly improves alkaline tolerance not only in sorghum but also in other monocot crops such as *Setaria italica* (foxtail millet), rice, wheat, and maize, demonstrating broad cross‐species application potential (Sun et al., 2023; Zhang et al., 2023).

#### Temperature and light stress

Extreme temperatures (chilling < 10°C; heat > 35°C) and high–light stress threaten crop survival by disrupting photosynthetic homeostasis and metabolic balance. As central signaling hubs, G‐protein α (Gα), β (Gβ), and γ (Gγ) subunits coordinately regulate stress tolerance via species‐specific mechanisms that mediate Ca^2+^ dynamics, lipid remodeling, and reactive oxygen species (ROS) scavenging.

Under chilling stress, COLD‐RESPONSIVE PROTEIN 1 (COLD1) in *japonica* rice interacts with Gα (RGA1) to activate Ca^2+^ influx and suppress excessive chilling‐induced ROS accumulation, thereby alleviating membrane lipid peroxidation and enhancing cold tolerance (Ma et al., 2015b; Luo et al., 2021). RGA1 is also involved in the COLD6‑dependent chilling tolerance response. The COLD6 associates with OSM1, a cold‐induced protein, under low‐temperature stress, thereby releasing RGA1 from the interaction complex. This process triggers an increase in intracellular cAMP levels, thereby enhancing chilling tolerance in rice. In cucumber, the Gγ subunit CsGG3.2 forms a complex with cysteine‐rich receptor‐like kinase CRK to promote CBF protein phosphorylation and stabilization, improving cold resistance (Bai et al., 2018).

The rice Gγ subunit TT2/GS3 negatively regulates heat tolerance by mediating Ca^2+^ signaling through interactions with Gα and Gβ, while simultaneously repressing wax biosynthesis. Moreover, recent studies have revealed that Gγ subunits enable precise decoding of heat‐triggered membrane signals into nuclear transcriptional responses via the TT2–DGK7–PA–MdPDE1–cAMP axis: High temperature activates diacylglycerol kinase DGK7, which catalyzes the production of phosphatidic acid (PA) as a lipid second messenger; TT2 negatively regulates PA production by inhibiting DGK7 phosphorylation at Ser^477^, preventing excessive signal amplification; and PA binds to the nuclear‐localized phosphodiesterase MdPDE1, promoting its nuclear import and cAMP hydrolysis, which downregulates the expression of heat‐responsive genes, including those encoding small heat‐shock proteins and ROS‐scavenging enzymes, thereby initiating the heat‐tolerance program (Kan et al., 2025).

High‐light stress induces ROS production and photoinhibition, with G protein showing species‐specific regulation. In Arabidopsis, GPA1 inhibits ABA signal transmission, thereby repressing the induced expression of APX2, a key photoprotective gene in bundle sheath cells. A loss‐of‐function mutation of *gpa1* leads to markedly enhanced antioxidant defense in plants under high‐light conditions (Galvez‐Valdivieso et al., 2009). Gβ subunit AGB1 directly binds to the photomorphogenic positive regulator HY5 and inhibits the DNA‐binding activity of HY5. Under blue light, the CRY1–AGB1 interaction releases the repression of *HY5* by *AGB1*, thereby activating downstream light‐responsive genes (Lian et al., 2018). *AGB1* and the red‐light receptor phyB antagonistically regulate the stability of *PIF3*. AGB1 promotes the accumulation of PIF3 protein, a negative regulator of photomorphogenesis, whereas phyB facilitates PIF3 degradation. The *AGB1* mutant *agb1‐2* shows a short hypocotyl and enhanced photomorphogenesis, consistent with increased sensitivity to light signals (Xu et al., 2019). Different from in Arabidopsis, the loss‐of‐function mutant of rice Gα subunit RGA1 *d1* enhances photoprotection through suppressed chlorophyll degradation and modified leaf angle (Ferrero‐Serrano et al., 2018). The *d1* mutant displays lower leaf temperature and a higher photochemical reflectance index, indicating stronger photoavoidance capacity and improved light‐harvesting efficiency. The expression levels of heat shock protein, chlorophyll catabolic enzyme, and ROS scavenging genes are downregulated in *d1*, whereas genes associated with photosystem components and chlorophyll biosynthesis are upregulated. These results demonstrate that the d1 mutant gains elevated photoprotection capacity and suffers less irreversible photoinhibitory damage under prolonged high light.

### Biotic stress resistance

Biotic stresses such as pathogens, pests, and other invasive organisms continuously threaten crop growth and survival, and plants have evolved a sophisticated two‐tiered defense system to cope with biotic threats. G proteins serve as pivotal signal regulators that integrate biotic stress cues and coordinate the activation of plant defense responses, which is crucial for maintaining the balance between plant growth and biotic stress resistance.

#### Physical barrier regulation

Physical barriers, primarily the cell wall and stomata, form the first line of defense against pathogen invasion, and their integrity is tightly modulated by G proteins.

In *Arabidopsis thaliana*, Gβ (AGB1) and Gγ (AGG1/AGG2) subunits are critical for cell wall reinforcement during pathogen challenge. Mutants lacking these subunits (*agb1‐1*, *agg1 agg2*) show impaired callose deposition and dysregulated expression of cell wall‐related genes when infected by *Plectosphaerella cucumerina*, leading to reduced xylose content and compromised disease resistance (Delgado‐Cerezo et al., 2012). Notably, the Gα mutant (*gpa1‐4*) does not show these defects, indicating subunit‐specific functions in cell wall regulation (Guo et al., 2025c). Additionally, G proteins interact with seven‐transmembrane domain (7TM) proteins to control the secretion of cellulose synthase (CESA), directly influencing cell wall mechanical strength and pathogen penetration resistance (McFarlane et al., 2021). Furthermore, GS3‐mediated regulation of cell wall synthesis in rice further synergistically enhances plant resistance to Brown Planthopper (BPH), a process regulated upstream by *miR159‐GAmybL2* (Shen et al., 2023).

Stomatal immunity, another key physical defense, is orchestrated by multiple G protein‐mediated pathways. Gα (GPA1) mediates pathogen‐induced stomatal closure to block pathogen entry (Assmann, 2002). Gβ (AGB1) forms a Gγ‐dependent complex with the receptor‐like kinase FERONIA (FER); in *agb1* or *agg1/2/3* mutants, FER's regulatory function on stomatal movement is significantly attenuated (Yu et al., 2018b). Furthermore, G proteins integrate hormone signals (e.g., abscisic acid) and ion channel activity to fine‐tune stomatal opening/closing dynamics during pathogen attack (Wang et al., 2001; Chen et al., 2004; Ge et al., 2015).

#### Innate immune responses

Upon pathogen perception, plants trigger a cascade of immune reactions—including reactive oxygen species (ROS) burst, Ca^2+^ influx, MAPK cascade activation, defense hormone synthesis, and defense gene expression—all of which are regulated by G proteins. ROS burst, an early immune hallmark, is dependent on G protein signaling. In Arabidopsis and *Nicotiana benthamiana*, loss of XLGs, Gβ, or Gγ subunits reduces pathogen‐induced ROS production (Liang et al., 2018; Xue et al., 2020; Zhao et al., 2022; Li et al., 2022d). Specifically, *Arabidopsis gpa1* mutants show decreased flagellin‐induced ROS levels, while mutants of the G protein regulator RGS1 show the opposite phenotype (Xue et al., 2020). In *N. benthamiana*, XLG3/4/5 couple with plasma membrane‐localized pattern recognition receptors (PRRs) to transduce immune signals, and their knockout increases susceptibility to pathogens (Li et al., 2022d). In tomatoes, overexpression of *XLG2* or *Gβγ* enhances salicylic acid (SA)‐dependent programmed cell death (PCD), a critical defense response against biotrophic pathogens (Karimian et al., 2024).

The MAPK cascade, a central immune signaling module, is also synergistically regulated by G proteins. In Arabidopsis, AGB1 and GPA1 cooperate to activate MAPK, while XLG1/2/3 directly modulate pathogen‐induced MAPK activation (Cheng et al., 2015; Yuan et al., 2017; Wang et al., 2023). XLG2 further serves nuclear regulatory functions: Upon pathogen perception, it is phosphorylated by cytoplasmic receptor‐like kinases (RLCKs), translocates to the nucleus, and inhibits the kinase activity of nuclear MLK1‐4 (negative immune regulators), thereby derepressing defense gene expression (Ma et al., 2022).

## MOLECULAR DESIGN STRATEGIES: G PROTEIN SIGNALING FOR UNCOUPLING CROP GROWTH–DEFENSE TRADE‐OFFS

High yield and stress resistance are two core goals of crop breeding. However, due to energy allocation, hormonal regulation, morphological contradictions, and other factors, they are mutually antagonistic and difficult to achieve simultaneously. Specifically, when plants face stress conditions (such as drought, high temperature, pests, and diseases), they reduce resources allocated to growth (cell division, photosynthesis, and biomass accumulation) and instead invest in stress resistance‐related metabolic synthesis (e.g., osmotic adjustment substances, antioxidants, and defense compounds) or structural construction, leading to decreased growth rate (Zhang et al., 2020); conversely, in favorable environments, plants focus on growth with reduced investment in stress resistance.

Long‐term breeding and research have shown that the trade‐off between growth and defense is a common phenomenon in crops. Under heat stress, a single amino acid mutation in the STKc_GSK3 kinase TaSG‐D1 enhances wheat heat tolerance, but this change is also found to reduce seed size, thereby decreasing yield potential (Cao et al., 2024). Under cold stress, plants synthesize antifreeze proteins and stabilize cell membrane structures to resist low temperatures through the ICE–CBF–COR core pathway, which is accompanied by the inhibition of growth‐related genes such as those involved in cell elongation, resulting in delayed growth and development (Liu et al., 2018a). Previous studies have shown that overexpression of *DREB1A* can significantly improve drought tolerance in various crops; however, excessive overexpression of *DREB1A* also causes severe dwarfism and delayed flowering in plants (Nakashima et al., 2009; Seo et al., 2009). Similar situations occur under salt or alkali stress (Yuan et al., 2024), flooding/hypoxic stress (Rankenberg et al., 2024), pathogens (Giolai and Laine, 2024), and pests (Liu et al., 2024).

Nevertheless, the emergence of a few exceptions seems to break the trade‐off between crop growth and defense, suggesting that there is a molecular regulatory mechanism in plants that integrates nutritional status and stress signals. Through precise resource allocation, plants can meet the needs of resisting adverse environments while maximizing the supply for growth. For example, the plasma membrane nitrate receptor protein NRT1.1B mediates the perception and integration of nitrate and ABA signals. When there is sufficient nitrogen supply in the environment, nitrogen occupies NRT1.1B, and plants tend to grow; when nitrogen is deficient, ABA binds to more NRT1.1B, and plants activate the stress resistance mode. The conserved dual function of NRT1.1B protein in higher plants allows plants to flexibly switch between “growth” and “stress resistance” modes according to the environment (Ma et al., 2025b). *ZmICE1a* increases grain weight by promoting starch synthesis in the central endosperm. Meanwhile, *ZmICE1a* inhibits fungal proliferation in the endosperm and indirectly regulates defense responses in the peripheral endosperm by negatively regulating defense genes such as antimicrobial peptides and the biosynthesis of IAA and JA. Similarly, several genes have recently been reported to play important roles in balancing growth and defense, including Arabidopsis AtPRMT3–RPS2B balancing growth and cold stress response (Wang et al., 2024b), rice OsSPX1/2 sensing phosphorus (Pi) concentration to balance rice growth and resistance (He et al., 2024), OsCPK18/OsCPK4 balancing rice growth and disease resistance (Li et al., 2022c), and ZmCRK5A–ZmSMH4–ZmKCH1 regulating drought resistance in maize while maintaining yield (Ma et al., 2025a).

As core signal integrators in plants, G proteins not only regulate key yield‐related traits but also mediate plant key responses to biotic and abiotic stresses, and are highly conserved in crops such as rice, wheat, maize, and sorghum. Therefore, the synergistic regulation of growth and stress resistance by G protein signaling is crucial for balancing crop yield and stress resistance, and also one of the core directions for the genetic improvement of high yield and stress resistance in crops in the future.

**Figure 3:**
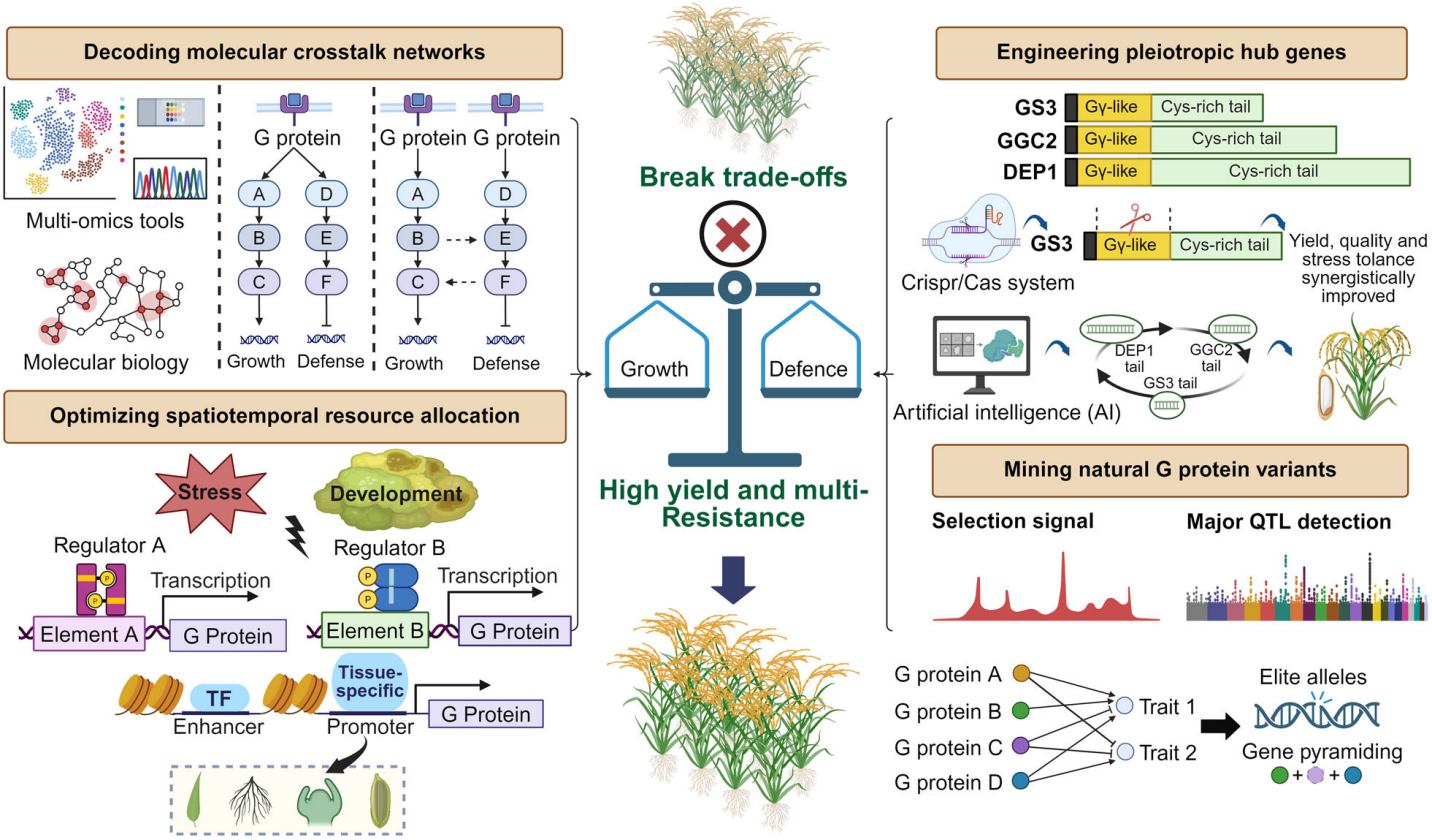
Strategies for uncoupling growth–defense trade‐offs by engineering G protein signaling Balancing defense and growth can be achieved through the following strategies: (1) Decoding Molecular Crosstalk Networks: Multi‐omics and molecular biology strategies enable the systematic dissection of key regulatory nodes and signaling crosstalk within G protein pathways governing plant growth and defense responses. G proteins concurrently activate two functionally distinct signaling cascades: one dedicated to growth regulation (A → B → C) and the other mediating defense responses (D → E → F), which are inherently antagonistic. By rewiring these downstream regulatory pathways into a unified signaling flow (A → B → E → F → C), it is possible to mitigate resource competition, reduce unnecessary energy consumption, and thereby decouple the growth–defense trade‐off in crops. (2) Optimizing spatiotemporal resource allocation: Environmental stress and developmental signals regulate the transcription of G protein‐encoding genes through distinct cis‐acting elements (Element A/B) and trans‐acting factors (Regulator A/B). The insertion of specific cis‐regulatory elements (enhancers and tissue‐specific promoters), in concert with transcription factors (TFs), determines the spatiotemporal expression patterns of G protein genes across different tissues (leaves, roots, meristems, and seeds), thereby enabling G protein signaling to coordinately regulate stress responses and growth and development in crops. (3) Engineering pleiotropic hub genes: Rice Gγ subunits (GS3, GGC2, and DEP1) contain an N‐terminal Gγ‐like domain and variable C‐terminal cysteine‐rich tails. CRISPR/Cas editing of the GS3 C‐terminal region synergistically improves yield, quality, and stress tolerance, while AI‐assisted sequence optimization accelerates the design of pleiotropic genes for multi‐trait crop improvement. (4) Mining natural G protein variants: Natural variants of G protein‐encoding genes (A–D) linked to target traits are identified through selection signal analysis and major QTL mapping. Dissection of genetic associations between G protein subunits and traits enables the selection of elite alleles for synergistic trait improvement, which can be combined via gene pyramiding to achieve simultaneous enhancement of target agronomic traits in crops.

The dual role of G proteins in yield and stress resistance makes them ideal targets for molecular breeding. Below, we synthesize translational strategies to optimize G protein function, emphasizing precision and cross‐species applicability (Figure 3).

### Decoding molecular crosstalk for trade‐off mechanism dissection

The inherent growth–defense trade‐off in crops is essentially determined by the pleiotropic cross‐regulation of G protein signaling. A single G protein subunit simultaneously participates in multiple pathways governing plant growth, development, and stress responses, resulting in widespread pleiotropic effects where any genetic modification leads to comprehensive phenotypic changes. This trade‐off is further shaped by complex signaling cascade interactions, metabolic flux competition, and transcriptional crosstalk, particularly the antagonism between JA/SA‐mediated defense pathways and GA‐dependent growth signaling, as well as resource partitioning between primary metabolism, such as photosynthate allocation, and the biosynthesis of secondary defensive metabolites. Fundamentally resolving this core dilemma requires the systematic dissection of the overall G protein regulatory network to separate independent modules for growth and defense, and eliminate the negative pleiotropic effects of G proteins. Successful application examples have been established in rice and other crops. In rice, precise manipulation of endogenous gibberellin (GA) levels via ATT1/ATT2 not only preserves desirable agronomic traits including semi‐dwarf architecture and high grain yield but also simultaneously enhances plant tolerance to alkaline stress and high‐temperature stress (Guo et al., 2025b). Targeting key crosstalk bottlenecks based on core plant hormone regulators including MYC2, DELLAs, and NPR1 enables the coordinated improvement of crop growth and stress tolerance. Future research should integrate systematic genetics, biochemistry, and multi‐omics strategies, including genomics, transcriptomics, proteomics, and metabolomics, to construct interaction networks between G protein subunits, hormones, transcription factors, kinases, and epigenetic regulators. This approach allows the precise identification of growth‐ and defense‐specific regulatory nodes, reduces pathway interference, clarifies global regulatory mechanisms, identifies key hub targets, predicts potential side effects, and optimizes molecular design breeding strategies. Artificial intelligence will further facilitate structural and functional prediction of G proteins; tools such as AlphaFold3 (Abramson et al., 2024; Fang et al., 2025b), molecular dynamics simulations, and molecular docking can accurately resolve the three‐dimensional conformation, interaction interfaces, and distinct functional domains of G proteins, enabling the clear separation of growth‐ and stress‐regulating domains at the molecular level and providing a solid theoretical basis for targeted modification (Sinha et al., 2022; Farhadi et al., 2025). AI‐assisted breeding has achieved promising preliminary results in several crops. For instance, the endogenous *NLA* gene in maize shows an inherent functional trade‐off: Enhanced cold tolerance is accompanied by reduced phosphorus uptake efficiency, while varieties with high phosphorus utilization are sensitive to low temperature (Liao et al., 2026). Combining AI‐driven protein structure design with gene editing technology, researchers can precisely modify the functional domains of NLA to decouple its cold stress‐regulatory module from the phosphorus transport module. This approach has successfully created new germplasm with strong cold tolerance and high phosphorus use efficiency, which delivers markedly improved yield under low‐temperature field conditions. Similarly, AI‐enabled tools also hold great potential to break the trade‐off between high yield and stress resistance by targeting G protein signaling pathways.

On this basis, G protein signaling pathways can be reconstructed and optimized using standardized biological parts, such as artificial promoters and synthetic transcription factors (Buson et al., 2024; Hancock et al., 2024). Environmentally responsive artificial signaling pathways can decouple growth and defense signaling, allowing crops to prioritize growth and yield under favorable conditions and rapidly activate defense responses under stress. Collectively, this innovative strategy fundamentally breaks the growth–defense trade‐off at the mechanistic level and provides a solid theoretical and technical foundation for G protein‐based precision breeding to achieve stable yield and high crop quality.

### Targeting pleiotropic hub genes for precision engineering of dual‐function modules

The functional characteristics of G proteins are fundamentally determined by their structural composition, among which the C‐terminal variable domain of Gγ subunits, the GTPase active domain of Gα subunits, and the interaction interface of Gβ subunits serve as the core sites for regulating functional polarity. The core advantage of molecular design targeting G protein subunits lies in its capacity to precisely manipulate signal transduction at the molecular level, which shows higher accuracy and predictability compared with conventional traditional breeding methods.

Modular structural modification of Gα, Gβ, and Gγ functional domains enables targeted regulation of specific downstream signaling pathways and effectively avoids unfavorable linkage drag in crop breeding. Notably, the functional specificity of plant Gγ subunits is predominantly determined by their C‐terminal variable “tail” region, while their conserved N‐terminal “head” domain is mainly responsible for binding to the Gβ subunit; this distinct structural feature makes the C‐terminal region of Gγ an ideal target for precise genetic modification. Evolutionary evidence shows that three typical rice Gγ proteins, DEP1, GGC2, and GS3, possess diverse regulatory functions due to evolutionary differentiation. Their ancestral genes originated from monocot σ duplication approximately 130 million years ago, and GGC2 was further derived from Poaceae ρ duplication around 70 million years ago, accompanied by the acquisition of unique tail repetitive sequences (Sun et al., 2025). Precise editing of the Gγ C‐terminus, such as targeted deletion or insertion of tail repetitive sequences, can directly switch the functional polarity of Gγ subunits (Sun et al., 2025). Based on advanced precision gene editing technologies including CRISPR/Cas9, base editing, and prime editing, directional modification of key G protein subunits can be customized to optimize a single agronomic function and effectively eliminate the antagonistic relationship between crop growth and defense responses. Multiple mature and emerging editing methods support diversified Gγ modification: CRISPR/Cas9‐mediated site‐directed mutagenesis can achieve gene knockout via frameshift mutations or large fragment deletions, while prime editing enables refined nucleotide substitutions and short fragment insertions or deletions (Yu et al., 2017; Chen et al., 2022); base editing, which fuses nCas9 or dCas9 with deaminases, achieves specific base conversion without inducing DNA double‐strand breaks and allows single amino acid substitution for in‐depth structure–function relationship research (Perrotta et al., 2025). For example, targeted modification can be performed on coding regions of G protein genes and cis‐acting elements in their promoters via prime editing, to fine‐tune G protein signaling activity without causing drastic phenotypic changes. In addition, it is also feasible to engineer downstream core targets in the regulatory cascade. For instance, we can conduct site‐directed modification on the phosphorylation sites of PIP2 regulated by AT1 (Zhang et al., 2023). These concrete technical strategies fully reflect the advantages of low‐interference precision editing, and we believe that this part will provide clearer guidance for relevant subsequent research.

Specifically, synthetic biology‐based modular functional domain editing can target key structures such as the Gγ C‐terminal tail and Gα active sites for deletion, insertion, or single amino acid replacement, thereby switching the regulatory polarity of subunits, for instance, converting yield‐negative regulatory loci into positive regulators while maintaining favorable stress resistance functions. Subunit chimera construction represents another innovative modification strategy, which fuses functional domains from different G protein subunits to generate novel chimeric proteins with synthetic functions (Rico and Höcker, 2013; Pillai et al., 2024). Given that Gγ consists of the N‐terminal conserved domain, the middle linker region, and the C‐terminal variable domain, exchanging and recombining these distinct domains can efficiently create chimeric Gγ proteins with customized regulatory capabilities (Zhao et al., 2025b). Meanwhile, traceless precise modification mediated by base editing and prime editing realizes accurate genetic improvement without DNA double‐strand breaks and exogenous gene insertion, avoiding transgenic risks and better meeting the biosafety and practical application requirements of commercial crop breeding. Collectively, these molecular modification strategies break the inherent functional limitations of natural G proteins at the structural level and act as core technical approaches to fundamentally resolve the growth–defense trade‐off in crops.

### Rewiring metabolic circuits for resource allocation optimization via synthetic biology

The trade‐off between crop growth and defense is not an overall antagonism at the whole‐plant level, but mainly a resource allocation conflict occurring at the spatial and temporal dimensions of specific organs or tissues. Precise spatiotemporal regulation of G protein expression patterns enables G proteins to specifically regulate yield formation in core growth organs such as panicles, grains, and shoot apical meristems, while focusing on enhancing stress tolerance in key defensive organs including roots, leaves, and stomata. This approach achieves optimal allocation of photosynthetic and metabolic resources at the whole‐plant level and fundamentally breaks the inherent trade‐off between growth and stress tolerance.

Previous studies have confirmed that rice G protein‐related genes show distinct tissue‐specific functional differentiation. For example, OsXLGs show distinct functional divergence. *OsXLG1* specifically mediates resistance to bacterial blight, while *OsXLG3* is essential for full blast resistance and dominates chitin‐triggered immune responses. The two genes exert opposing effects on yield traits: Knocking out OsXLG1 improves panicle morphology and grain weight with yield‐boosting potential, whereas mutation of OsXLG3 impairs vegetative growth, panicle development, and grain filling (Zhao et al., 2022). OsXLG3 is simultaneously involved in grain development, plant growth, and stress defense, achieving a dynamic balance between growth and resistance through multi‐organ coordination. Its functional transformation only adjusts the priority of plant resource allocation and does not cause whole‐plant growth disorders. Under non‐stress conditions, OsXLG3 regulates nutrient transport to grains and promotes grain filling; under pathogen stress, it cooperates with other XLG family members to prioritize immune defense responses over growth processes, coordinating biomass accumulation and stress tolerance to maintain normal plant growth and survival under adverse conditions (Cui et al., 2020; Ak et al., 2022). Another typical regulatory module is the *OsPLATZ1–OsGRF4–DEP1* pathway, a key molecular module controlling rice grain shape. The core transcriptional activator OsPLATZ1 interacts with multiple GRF proteins and binds to the promoter region of *DEP1* to exert regulatory functions, but OsPLATZ1 shows opposite regulatory effects on grain number per panicle and grain length, forming an inherent trait trade‐off. Precise modification of the promoter region of *OsPLATZ1*, an upstream regulator of *DEP1*, can optimize gene expression patterns, increase G protein accumulation in grains, and synergistically improve panicle grain number and grain morphology, providing an excellent breeding target for cultivating high‐yield and high‐quality rice varieties (Chen et al., 2025b).

Based on this physiological regulatory logic, three precise regulation strategies can resolve the growth–defense trade‐off. First, tissue‐specific promoter‐driven regulation uses organ‐specific promoters for grains, panicles, and roots to realize targeted expression of G protein subunits, enhancing grain filling and grain weight regulation in grains and strengthening alkaline tolerance, drought resistance, and disease defense responses in leaves and roots. Second, stress‐inducible dynamic regulation adopts promoters induced by drought, salinity, and pathogens to trigger G protein‐mediated defense signaling only under stress conditions, while shutting down defense metabolic consumption and fully supporting crop growth under normal conditions to achieve environment‐responsive functional switching. Third, developmental stage‐specific precise temporal regulation combines stage‐specific regulatory elements for vegetative and reproductive growth, enabling G proteins to optimize plant architecture during the vegetative stage, improve panicle grain number and 1,000‐grain weight during the reproductive stage, and rapidly activate stress defense pathways under adverse conditions.

These regulatory strategies conform to the natural energy metabolism and resource allocation rules of plants, and fundamentally resolve the crop growth–defense trade‐off at the mechanistic level. Combined with tissue‐specific promoters, stress‐inducible promoters, and precise promoter editing technologies, the spatiotemporal expression of G protein signaling can be finely tuned to efficiently create new crop germplasms with coordinately improved yield, quality, and stress resistance, representing the most promising molecular design strategy for practical field breeding applications.

### Mining natural G protein variants for balanced yield and stress resistance

Growing evidence indicates that natural variation represents a precious and optimal breeding resource for breaking the growth–defense trade‐off. These elite genetic variations have been naturally selected and retained during crop domestication and long‐term evolutionary adaptation, and they can achieve the coordinated improvement of high yield and stress tolerance merely through genomic selection and molecular pyramiding without artificial gene editing, making them the safest and most efficient breeding strategy for practical application.

Crop natural variations are derived from wild relatives and continuously shaped by local adaptation and long‐term artificial selection during domestication (Liang et al., 2021). Focusing on the selection signals of G protein subunits and mining favorable natural variations within the G protein family have become a feasible strategy to break the inherent growth–defense trade‐off and realize the synergistic improvement of crop yield and stress resistance. Most natural variations of G protein subunits are predominantly enriched in the C‐terminal regulatory domain of Gγ subunits and the tissue‐specific expression elements of XLG subunits. Precise dissection of the functional differentiation of G proteins in different organs can effectively eliminate the adverse chain reaction of growth inhibition accompanied by defense activation. For instance, natural allelic variants of the rice Gγ subunit gene *GS3*, including *GS3‐1*, *GS3‐2*, and *GS3‐3*, alter the C‐terminal integrity of GS3 protein, in which the full‐length C‐terminal allele mediates ubiquitin‐dependent Gγ protein degradation (Yang et al., 2021), whereas the loss‐of‐function *GS3‐3* variant eliminates full‐length GS3 protein accumulation and produces longer grains (Mao et al., 2010). These variations not only relieve the inhibition of grain cell proliferation to significantly increase grain length and yield but also promote cuticular wax accumulation in leaves and enhance alkali tolerance in roots, thereby achieving an ideal coordination between high yield and multiple stress resistance (Kan et al., 2022; Zhang et al., 2023). In Arabidopsis, natural *XLG2* variants modify the phosphorylation sites of the nuclear localization signal, enabling XLG2 to specifically accumulate in leaf cell nuclei to activate immune defense responses upon pathogen infection without migrating to floral organs and interfering with reproductive growth, thus breaking the traditional trade‐off whereby defense activation inevitably suppresses plant reproduction (Heo et al., 2012; Ma et al., 2022). The core advantage of these natural variations is that they do not completely eliminate G protein functions but fine‐tune protein activity and expression patterns through domain‐specific mutations and subtle regulatory adjustments, achieving a natural win–win balance between growth and defense and providing safe and elite targets for molecular breeding.

To efficiently explore and utilize these valuable genetic resources, three major technical routes can be adopted: Core variation site mining, evolutionary locus selection, and elite allele pyramiding. Core variation mining focuses on functional hotspot regions, such as the Gγ C‐terminal regulatory domain and *XLG* tissue‐specific expression elements, to screen superior haplotypes with both high‐yield and stress‐resistant advantages, including rice *GS3‐3*, dominant *DEP1* alleles, and sorghum *AT1* deletion variations. Evolutionary locus selection analyzes the domestication trajectories of G protein subunits in gramineous crops such as rice, maize, wheat, and sorghum, and identifies evolutionarily conserved superior variations for wide‐adaptability genetic improvement. Elite allele pyramiding utilizes marker‐assisted selection to combine high‐yield G protein alleles such as *dep1* and *gs3* with stress‐resistant alleles including mutated *AT1*, facilitating the breeding of new crop varieties with high yield, superior quality, and broad‐spectrum multiple resistance.

At present, molecular design and application of G protein subunits have shown enormous breeding potential in crops. *DEP1* is one of the most widely utilized high‐yield genes in rice breeding, and its controlled erect panicle architecture has been globally applied for more than a century (Xu et al., 2016). The Gγ subunit edited *dep1‐cys* mutant not only forms dense and erect high‐yield panicles but also enhances sheath blight resistance and relieves high‐nitrogen‐induced susceptibility; furthermore, *DEP1* can be pyramided with *IPA1*, *Gn1a*, and *GS3* to develop super hybrid rice utilizing indica–japonica heterosis and further optimize yield components (Li et al., 2022b). In tomato, editing the Gγ subunit *GGC1* enhances photosynthetic efficiency, increases fruit weight and fruit number, and promotes the accumulation of nutritional components such as carotenoids. These successful cases fully demonstrate the broad application prospects and great practical value of G protein molecular design (Liang et al., 2025b). Overall, this natural variation‐based breeding strategy takes advantage of natural evolutionary domestication, avoids the potential risks of artificial genetic modification, and represents the most easily implemented and industrialized approach for modern crop molecular breeding.

## CONCLUSION AND PERSPECTIVE

G proteins are conserved signal integrators that coordinate crop yield and stress resistance via modular signaling networks, with subunit‐specific functions shaped by structural variations (e.g., Gγ C‐terminal domains) and species‐specific metabolic pathways. However, research on G protein signaling still needs to be further uncovered. For example, how G proteins perceive stress signals is mostly unknown. Under osmotic stress, early signal perception before massive ABA accumulation can induce liquid–liquid phase separation (LLPS) of plant proteins. For instance, the nuclear protein SAM8 can directly sense changes in cellular water potential and rapidly form condensates, and this process is independent of the canonical osmotic stress pathway (Wang et al., 2026). The plant‐specific GBPL family GTPases can form defense‐activated condensates through LLPS to regulate the expression of immunity‐related genes; yet, phase separation of canonical heterotrimeric G proteins has not been reported (Huang et al., 2021). Studies have found that canonical G proteins achieve non‐canonical activation via phosphorylation at the conserved site of the Gα subunit (Jia et al., 2025). Follow‐up research on how G protein signaling directly perceives stress signals may help break the limitation of upstream signal sensing of G proteins and resolve the trade‐off between plant growth and stress tolerance in breeding practices. Core mechanisms include competitive binding between Gγ variants, synergistic interaction with TFs, and cross‐pathway integration (hormone signaling, ROS homeostasis, and epigenetic regulation). Translational strategies—systems biology‐driven network dissection, precision gene editing, natural variation mining, and tissue‐specific regulation—enable targeted optimization of G protein function to break growth–defense trade‐offs.

Despite progress, gaps remain: Limited research on G protein pathways in maize, wheat, and sorghum; incomplete understanding of multi‐stress responses; and regulatory barriers for gene‐edited crops. Future directions should focus on (i) dissecting conserved and species‐specific G protein functions in staple crops; (ii) developing precision editing tools for minimal genetic modification; (iii) integrating AI and machine learning to predict trade‐off outcomes; and (iv) validating synthetic biology approaches under field conditions. By addressing these challenges, G protein signaling will pave the way for climate‐resilient crops with balanced yield and stress tolerance, ensuring food security in a changing environment.

## CONFLICTS OF INTEREST

The authors declare no conflicts of interest.

## AUTHOR CONTRIBUTIONS

P.X. designed and developed the framework. H.L., Z.Z., and F.L. wrote the manuscript. H.W. and Z.H. checked the literature review. P.X., X.D., and S.T. contributed to the critical review and editing of the manuscript. All authors have read and agreed to the published version of the manuscript.

## References

[jipb70351-bib-0001] Abramson, J. , Adler, J. , Dunger, J. , Evans, R. , Green, T. , Pritzel, A. , Ronneberger, O. , Willmore, L. , Ballard, A.J. , Bambrick, J. , et al. (2024). Accurate structure prediction of biomolecular interactions with AlphaFold 3. Nature 630: 493–500. 38718835 10.1038/s41586-024-07487-w PMC11168924

[jipb70351-bib-0002] Ak, B. , Ty, W. , D, U. , R, P. , Jb, M. , Am, J. , and Ak, B. (2022). Novel mutant alleles reveal a role of the extra‐large G protein in rice grain filling, panicle architecture, plant growth, and disease resistance. Front. Plant Sci. 12: 782960. 35046975 10.3389/fpls.2021.782960 PMC8761985

[jipb70351-bib-0003] Assmann, S.M. (2002). Heterotrimeric and unconventional GTP binding proteins in plant cell signaling. Plant Cell 14: S355–S373. 12045288 10.1105/tpc.001792 PMC151266

[jipb70351-bib-0004] Bai, L. , Liu, Y. , Mu, Y. , Anwar, A. , He, C. , Yan, Y. , Li, Y. , and Yu, X. (2018). Heterotrimeric G‐protein γ subunit CsGG3.2 positively regulates the expression of *CBF* genes and chilling tolerance in cucumber. Front. Plant Sci. 9: 488. 29719547 10.3389/fpls.2018.00488 PMC5913349

[jipb70351-bib-0005] Biswas, S. , Islam, M.dN. , Sarker, S. , Tuteja, N. , and Seraj, Z.I. (2019). Overexpression of heterotrimeric G protein beta subunit gene (*OsRGB1*) confers both heat and salinity stress tolerance in rice. Plant Physiol. Biochem. 144: 334–344. 31622936 10.1016/j.plaphy.2019.10.005

[jipb70351-bib-0006] Bommert, P. , Je, B.I. , Goldshmidt, A. , and Jackson, D. (2013). The maize Gα gene *COMPACT PLANT2* functions in CLAVATA signalling to control shoot meristem size. Nature 502: 555–558. 24025774 10.1038/nature12583

[jipb70351-bib-0007] Brunetti, S.C. , Arseneault, M.K.M. , Wright, J.A. , Wang, Z. , Ehdaeivand, M.‐R. , Lowden, M.J. , Rivoal, J. , Khalil, H.B. , Garg, G. , and Gulick, P.J. (2021). The stress induced caleosin, RD20/CLO3, acts as a negative regulator of GPA1 in Arabidopsis. Plant Mol. Biol. 107: 159–175. 34599731 10.1007/s11103-021-01189-x

[jipb70351-bib-0008] Buson, F. , Gao, Y. , and Wang, B. (2024). Genetic parts and enabling tools for biocircuit design. ACS Synth. Biol. 13: 697–713. 38427821 10.1021/acssynbio.3c00691

[jipb70351-bib-0009] Cantos, C.F. , dePamphilis, C.W. , and Assmann, S.M. (2023). Extra‐large G proteins have extra‐large effects on agronomic traits and stress tolerance in maize and rice. Trends Plant Sci. 28: 1033–1044. 37156701 10.1016/j.tplants.2023.04.005 PMC10524845

[jipb70351-bib-0010] Cao, J. , Qin, Z. , Cui, G. , Chen, Z. , Cheng, X. , Peng, H. , Yao, Y. , Hu, Z. , Guo, W. , Ni, Z. , et al. (2024). Natural variation of STKc_GSK3 kinase TaSG‐D1 contributes to heat stress tolerance in Indian dwarf wheat. Nat. Commun. 15: 2097. 38453935 10.1038/s41467-024-46419-0 PMC10920922

[jipb70351-bib-0011] Chen, H. , Yu, Z. , Wang, X. , Lu, J. , Li, X. , Gao, J. , Li, F. , Xu, H. , Xu, Q. , Zhang, W. , et al. (2025a). NITRATE TRANSPORTER1/PEPTIDE TRANSPORTER FAMILY4 interacts with DENSE AND ERECT PANICLE1 and regulates grain number in rice. Plant Physiol. 198: 210. 10.1093/plphys/kiaf210 40489297

[jipb70351-bib-0012] Chen, S. , Xu, C. , Tan, Y. , Zhang, S. , Huang, Y. , Yang, Q. , Zhang, Z. , Li, F. , Wang, L. , Li, Z. , et al. (2025b). The *OsPLATZ1*‐*OsGRF4*‐*DEP1* regulatory pathway promotes grain length in rice. J. Integr. Plant Biol. 67: 2594–2608. 40747874 10.1111/jipb.70009

[jipb70351-bib-0013] Chen, W. , She, W. , Li, A. , Zhai, C. , and Ma, L. (2022). Site‐directed mutagenesis method mediated by Cas9. Methods Mol. Biol. 2461: 165–174. 35727450 10.1007/978-1-0716-2152-3_11

[jipb70351-bib-0014] Chen, Y.L. , Huang, R. , Xiao, Y.M. , Lü, P. , Chen, J. , and Wang, X.C. (2004). Extracellular calmodulin‐induced stomatal closure is mediated by heterotrimeric G protein and H_2_O_2_ . Plant Physiol. 136: 4096–4103. 15557100 10.1104/pp.104.047837 PMC535840

[jipb70351-bib-0015] Cheng, Z. , Li, J.F. , Niu, Y. , Zhang, X.C. , Woody, O.Z. , Xiong, Y. , Djonović, S. , Millet, Y. , Bush, J. , McConkey, B.J. , et al. (2015). Pathogen‐secreted proteases activate a novel plant immune pathway. Nature 521: 213–216. 25731164 10.1038/nature14243 PMC4433409

[jipb70351-bib-0016] Choudhury, S.R. , and Pandey, S. (2013). Specific subunits of heterotrimeric G proteins play important roles during nodulation in soybean. Plant Physiol. 162: 522–533. 23569109 10.1104/pp.113.215400 PMC3641229

[jipb70351-bib-0017] Colaneri, A.C. , Tunc‐Ozdemir, M. , Huang, J.P. , and Jones, A.M. (2014). Growth attenuation under saline stress is mediated by the heterotrimeric G protein complex. BMC Plant Biol. 14: 129. 24884438 10.1186/1471-2229-14-129 PMC4061919

[jipb70351-bib-0018] Cui, Y. , Jiang, N. , Xu, Z. , and Xu, Q. (2020). Heterotrimeric G protein are involved in the regulation of multiple agronomic traits and stress tolerance in rice. BMC Plant Biol. 20: 90. 32111163 10.1186/s12870-020-2289-6 PMC7048073

[jipb70351-bib-0161] Delgado‐Cerezo, M. , Sánchez‐Rodríguez, C. , Escudero, V. , Miedes, E ., Fernández, P.V. , Jordá, L. , Hernández‐Blanco, C. , Sánchez‐Vallet, A. , Bednarek, P. , Schulze‐Lefert, P. , et al. (2012). Arabidopsis heterotrimeric G‐protein regulates cell wall defense and resistance to necrotrophic fungi. Mol. Plant 5: 98–114. 21980142 10.1093/mp/ssr082

[jipb70351-bib-0019] Fang, J. , Chun, Y. , Zhang, F. , Guo, T. , Ren, M. , Zhao, J. , Yuan, S. , Wang, W. , Li, Y. , and Li, X. (2025a). A novel *OsMPK6*‐*OsMADS47*‐*PPKL1/3* module controls grain shape and yield in rice. Adv. Sci. 12: e01946. 10.1002/advs.202501946 PMC12376620 40470766

[jipb70351-bib-0020] Fang, Z. , Ran, H. , Zhang, Y. , Chen, C. , Lin, P. , Zhang, X. , and Wu, M. (2025b). AlphaFold 3: An unprecedent opportunity for fundamental research and drug development. Precis. Clin. Med. 8: pbaf015. 40799364 10.1093/pcmedi/pbaf015 PMC12342994

[jipb70351-bib-0021] Farhadi, B. , Beygisangchin, M. , Ghamari, N. , Jakmunee, J. , and Tang, T. (2025). Molecular dynamics simulations of proteins: An in‐depth review of computational strategies, structural insights, and their role in medicinal chemistry and drug development. Biol. Cybern. 119: 28. 41003729 10.1007/s00422-025-01026-0

[jipb70351-bib-0022] Ferrero‐Serrano, Á. , and Assmann, S.M. (2016). The α‐subunit of the rice heterotrimeric G protein, RGA1, regulates drought tolerance during the vegetative phase in the dwarf rice mutant *d1* . J. Exp. Bot. 67: 3433–3443. 27194741 10.1093/jxb/erw183 PMC4892740

[jipb70351-bib-0023] Ferrero‐Serrano, Á. , Su, Z. , and Assmann, S.M. (2018). Illuminating the role of the Gα heterotrimeric G protein subunit, RGA1, in regulating photoprotection and photoavoidance in rice. Plant Cell Envir 41: 451–468. 10.1111/pce.13113 29216416

[jipb70351-bib-0024] Fujisawa, Y. , Kato, T. , Ohki, S. , Ishikawa, A. , Kitano, H. , Sasaki, T. , Asahi, T. , and Iwasaki, Y. (1999). Suppression of the heterotrimeric G protein causes abnormal morphology, including dwarfism, in rice. Proc. Natl. Acad. Sci. U.S.A. 96: 7575–7580. 10377457 10.1073/pnas.96.13.7575 PMC22128

[jipb70351-bib-0025] Galvez‐Valdivieso, G. , Fryer, M.J. , Lawson, T. , Slattery, K. , Truman, W. , Smirnoff, N. , Asami, T. , Davies, W.J. , Jones, A.M. , Baker, N.R. , et al. (2009). The high light response in Arabidopsis involves ABA signaling between vascular and bundle sheath cells. Plant Cell 21: 2143–2162. 19638476 10.1105/tpc.108.061507 PMC2729609

[jipb70351-bib-0026] Gao, M. , Hao, Z. , Ning, Y. , and He, Z. (2024). Revisiting growth–defence trade‐offs and breeding strategies in crops. Plant Biotechnol. J. 22: 1198–1205. 38410834 10.1111/pbi.14258 PMC11022801

[jipb70351-bib-0027] Ge, X.M. , Cai, H.L. , Lei, X. , Zhou, X. , Yue, M. , and He, J.M. (2015). Heterotrimeric G protein mediates ethylene‐induced stomatal closure via hydrogen peroxide synthesis in Arabidopsis. Plant J. 82: 138–150. 25704455 10.1111/tpj.12799

[jipb70351-bib-0028] Giolai, M. , and Laine, A.L. (2024). A trade‐off between investment in molecular defense repertoires and growth in plants. Science 386: 677–680. 39509497 10.1126/science.adn2779

[jipb70351-bib-0029] Guo, J.F. , Zhou, H. , Hu, Z.‐R. , Yang, Y.L. , Wang, W.B. , Zhang, Y.R. , Li, X. , Mulati, N. , Li, Y.X. , Wu, L. , et al. (2025c). The Arabidopsis heterotrimeric G protein α subunit binds to and inhibits the inward rectifying potassium channel KAT1. Plant Sci. 352: 112363. 39710151 10.1016/j.plantsci.2024.112363

[jipb70351-bib-0030] Guo, S. , Wang, Y. , Wu, J. , Zhou, X. , and Gao, H. (2025a). Heterotrimeric G‐proteins: Multi‐dimensional regulation in plant growth, development and abiotic stress responses. Stress Biol. 5: 3.

[jipb70351-bib-0031] Guo, S.Q. , Chen, Y.X. , Ju, Y.L. , Pan, C.Y. , Shan, J.X. , Ye, W.W. , Dong, N.Q. , Kan, Y. , Yang, Y.B. , Zhao, H.Y. , et al. (2025b). Fine‐tuning gibberellin improves rice alkali–thermal tolerance and yield. Nature 639: 162–171. 39880957 10.1038/s41586-024-08486-7

[jipb70351-bib-0032] Han, L. , Huang, Y. , Li, C. , Tian, D. , She, D. , Li, M. , Wang, Z. , Chen, J. , Liu, L. , Wang, S. , et al. (2024). Heterotrimeric Gα‐subunit regulates flower and fruit development in CLAVATA signaling pathway in cucumber. Hortic. Res. 11: uhae110. 38898960 10.1093/hr/uhae110 PMC11186068

[jipb70351-bib-0033] Hancock, B. , Tobias, A.V. , Leung, J.K. , Markina‐Khusid, A. , and Salit, M. (2024). Modularization and systems engineering in synthetic biology: A formal architecture modeling approach using SysML. In 2024 IEEE International Systems Conference (SysCon), 1–3.

[jipb70351-bib-0034] He, Y. , Zhao, Y. , Hu, J. , Wang, L. , Li, L. , Zhang, X. , Zhou, Z. , Chen, L. , Wang, H. , Wang, J. , et al. (2024). The OsBZR1–OsSPX1/2 module fine‐tunes the growth–immunity trade‐off in adaptation to phosphate availability in rice. Mol. Plant 17: 258–276. 38069474 10.1016/j.molp.2023.12.003

[jipb70351-bib-0035] Heo, J.B. , Sung, S. , and Assmann, S.M. (2012). Ca^2+^‐dependent GTPase, extra‐large G protein 2 (XLG2), promotes activation of DNA‐binding protein related to vernalization 1 (RTV1), leading to activation of floral integrator genes and early flowering in Arabidopsis. J. Biol. Chem. 287: 8242–8253. 22232549 10.1074/jbc.M111.317412 PMC3318724

[jipb70351-bib-0036] Huang, H. , and Qian, Q. (2017). Progress in genetic research of rice grain shape and breeding achievements of long‐grain shape and good quality japonica rice. Chin. J. Rice Sci. 31: 665.

[jipb70351-bib-0037] Huang, J. , Gao, L. , Luo, S. , Liu, K. , Qing, D. , Pan, Y. , Dai, G. , Deng, G. , and Zhu, C. (2022). The genetic editing of GS3 via CRISPR/Cas9 accelerates the breeding of three‐line hybrid rice with superior yield and grain quality. Mol. Breed. 42: 22. 37309462 10.1007/s11032-022-01290-z PMC10248666

[jipb70351-bib-0038] Huang, S. , Zhu, S. , Kumar, P. , and MacMicking, J.D. (2021). A phase‐separated nuclear GBPL circuit controls immunity in plants. Nature 594: 424–429. 34040255 10.1038/s41586-021-03572-6 PMC8478157

[jipb70351-bib-0039] Huang, X. , Qian, Q. , Liu, Z. , Sun, H. , He, S. , Luo, D. , Xia, G. , Chu, C. , Li, J. , and Fu, X. (2009). Natural variation at the DEP1 locus enhances grain yield in rice. Nat. Genet. 41: 494–497. 19305410 10.1038/ng.352

[jipb70351-bib-0040] Hultgren, A. , Carleton, T. , Delgado, M. , Gergel, D.R. , Greenstone, M. , Houser, T. , Hsiang, S. , Jina, A. , Kopp, R.E. , Malevich, S.B. , et al. (2025). Impacts of climate change on global agriculture accounting for adaptation. Nature 642: 644–652. 40533541 10.1038/s41586-025-09085-w PMC12176627

[jipb70351-bib-0041] Jangam, A.P. , Pathak, R.R. , and Raghuram, N. (2016). Microarray analysis of rice *d1* (*RGA1*) mutant reveals the potential role of G‐protein alpha subunit in regulating multiple abiotic stresses such as drought, salinity, heat, and cold. Front. Plant Sci. 7: 11. 26858735 10.3389/fpls.2016.00011 PMC4729950

[jipb70351-bib-0042] Jia, H. , Hewitt, N. , Jordá, L. , Abramyan, T.M. , Tolliver, J. , Jones, J.L. , Nomura, K. , Yang, J. , He, S.‐Y. , Tropsha, A. , et al. (2025). Phosphorylation‐activated G protein signaling stabilizes TCP14 and JAZ3 to repress JA signaling and enhance plant immunity. Mol. Plant 18: 1171–1192. 40509595 10.1016/j.molp.2025.06.004 PMC12817259

[jipb70351-bib-0043] Kan, Y. , Mu, X.R. , Qu, F. , Dai, Z.A. , Gao, J. , Liu, N.J. , Li, S. , Shan, J.X. , Ye, W.W. , Dong, N.Q. , et al. (2025). A stepwise decoding mechanism for heat sensing in plants connects lipid remodeling to a nuclear signaling cascade. Cell 188: 7378–7396. 41338194 10.1016/j.cell.2025.11.003

[jipb70351-bib-0044] Kan, Y. , Mu, X.R. , Zhang, H. , Gao, J. , Shan, J.X. , Ye, W.W. , and Lin, H.X. (2022). TT2 controls rice thermotolerance through SCT1‐dependent alteration of wax biosynthesis. Nat. Plants 8: 53–67. 34992240 10.1038/s41477-021-01039-0

[jipb70351-bib-0045] Karimian, P. , Trusov, Y. , Botella, J.R. , Karimian, P. , Trusov, Y. , and Botella, J.R. (2024). Conserved role of heterotrimeric g proteins in plant defense and cell death progression. Genes 15: 115. 38255003 10.3390/genes15010115 PMC10815853

[jipb70351-bib-0046] Kumar, R. , and Bisht, N.C. (2020). Heterotrimeric Gα subunit regulates plant architecture, organ size and seed weight in the oilseed *Brassica juncea* . Plant Mol. Biol. 104: 549–560. 32875468 10.1007/s11103-020-01060-5

[jipb70351-bib-0047] Li, A. , Hao, C. , Wang, Z. , Geng, S. , Jia, M. , Wang, F. , Han, X. , Kong, X. , Yin, L. , Tao, S. , et al. (2022a). Wheat breeding history reveals synergistic selection of pleiotropic genomic sites for plant architecture and grain yield. Mol. Plant 15: 504–519. 35026438 10.1016/j.molp.2022.01.004

[jipb70351-bib-0048] Li, H. , Zhang, Y. , Wu, C. , Bi, J. , Chen, Y. , Jiang, C. , Cui, M. , Chen, Y. , Hou, X. , Yuan, M. , et al. (2022c). Fine‐tuning OsCPK18/OsCPK4 activity via genome editing of phosphorylation motif improves rice yield and immunity. Plant Biotechnol. J. 20: 2258–2271. 35984919 10.1111/pbi.13905 PMC9674324

[jipb70351-bib-0049] Li, M. , Pan, X. , and Li, H. (2022b). Pyramiding of *gn1a*, *gs3*, and *ipa1* exhibits complementary and additive effects on rice yield. Int. J. Mol. Sci. 23: 12478. 36293334 10.3390/ijms232012478 PMC9604080

[jipb70351-bib-0050] Li, Q. , Yang, X. , Bai, G. , Warburton, M.L. , Mahuku, G. , Gore, M. , Dai, J. , Li, J. , and Yan, J. (2010). Cloning and characterization of a putative GS3 ortholog involved in maize kernel development. Theor. Appl. Genet. 120: 753–763. 19898828 10.1007/s00122-009-1196-x

[jipb70351-bib-0051] Li, S. , Zhao, Z. , Liu, T. , Zhang, J. , Xing, X. , Feng, M. , Liu, X. , Luo, S. , Dong, K. , Wang, J. , et al. (2025a). The G‐protein γ subunit DEP1 facilitates brassinosteroid signaling in rice via a MYB–bHLH–ARF module. Plant Cell 37: koaf122. 40398925 10.1093/plcell/koaf122 PMC12124404

[jipb70351-bib-0052] Li, Y. , Ning, Q. , Zhao, R. , Liu, D. , Li, N. , Xiong, Q. , Sun, Q. , Du, Y. , Mao, R. , Zhan, J. , et al. (2025b). Coordinated improvement of maize grain yield and protein quality by the ZmMADS8‐ZmMADS47‐O2 module and a G protein gamma subunit. Crop. J. 13: 805–817.

[jipb70351-bib-0053] Li, Y. , Zhang, Q. , Gong, L. , Kong, J. , Wang, X. , Xu, G. , Chen, X. , Dou, D. , and Liang, X. (2022d). Extra‐large G proteins regulate disease resistance by directly coupling to immune receptors in *Nicotiana benthamiana* . Phytopathol. Res. 4: 49.

[jipb70351-bib-0054] Li, Y. , Zhao, W. , Peng, X. , Liang, Y. , Xue, R. , Liu, J. , Xu, Z. , Cao, D. , and Liu, B. (2026). *TaGα* knockout in wheat causes early heading and short organ length, with dose‐dependent effects through various pathways. Theor. Appl. Genet. 139: 131. 42026322 10.1007/s00122-026-05239-0

[jipb70351-bib-0055] Lian, H. , Xu, P. , He, S. , Wu, J. , Pan, J. , Wang, W. , Xu, F. , Wang, S. , Pan, J. , Huang, J. , et al. (2018). Photoexcited CRYPTOCHROME 1 interacts directly with G‐protein β subunit AGB1 to regulate the DNA‐binding activity of HY5 and photomorphogenesis in Arabidopsis. Mol. Plant 11: 1248–1263. 30176372 10.1016/j.molp.2018.08.004

[jipb70351-bib-0056] Liang, X. , Li, Y. , Zai, W. , Huang, S. , and Shi, K. (2025a). G‐proteins at the crossroads of signaling and stress tolerance in horticultural crops. Hortic. Plant J. 11: 1744–1760.

[jipb70351-bib-0057] Liang, X. , Ma, M. , Zhou, Z. , Wang, J. , Yang, X. , Rao, S. , Bi, G. , Li, L. , Zhang, X. , Chai, J. , et al. (2018). Ligand‐triggered de‐repression of Arabidopsis heterotrimeric G proteins coupled to immune receptor kinases. Cell Res. 28: 529–543. 29545645 10.1038/s41422-018-0027-5 PMC5951851

[jipb70351-bib-0058] Liang, X. , Wang, A. , Yue, C. , Ding, S. , Zhou, S. , Yu, Q. , Zhang, X. , Luo, Q. , Li, Y. , Wu, Y. , et al. (2025b). Functional divergence of G protein γ subunits drives plant vigor and improvement through the CPK28‐PIP1;2 pathway in tomato. Cell Rep. 44: 116253. 40913765 10.1016/j.celrep.2025.116253

[jipb70351-bib-0059] Liang, Y. , Liu, H.‐J. , Yan, J. , and Tian, F. (2021). Natural variation in crops: Realized understanding, continuing promise. Annu. Rev. Plant Biol. 72: 357–385. 33481630 10.1146/annurev-arplant-080720-090632

[jipb70351-bib-0060] Liao, H. , Zhao, X. , Ren, K. , Guo, L. , Li, Z. , Liu, Z. , Zhang, X. , Su, T. , Fu, D. , Zhang, Z. , et al. (2026). Rewiring an E3 ligase enhances cold resilience and phosphate use in maize. Nature 653: 831–839. 41741638 10.1038/s41586-026-10142-1

[jipb70351-bib-0061] Liu, C. , Mao, B. , Yuan, D. , Chu, C. , and Duan, M. (2022). Salt tolerance in rice: Physiological responses and molecular mechanisms. Crop. J. 10: 13–25.

[jipb70351-bib-0062] Liu, C. , Xu, Y. , Feng, Y. , Long, D. , Cao, B. , Xiang, Z. , and Zhao, A. (2019). Ectopic expression of mulberry G‐proteins alters drought and salt stress tolerance in tobacco. Int. J. Mol. Sci. 20: 89. 10.3390/ijms20010089 PMC6337368 30587818

[jipb70351-bib-0063] Liu, J. , Li, L. , Xiong, Z. , Robert, C.A.M. , Li, B. , He, S. , Chen, W. , Bi, J. , Zhai, G. , Guo, S. , et al. (2024). Trade‐offs between the accumulation of cuticular wax and jasmonic acid‐mediated herbivory resistance in maize. J. Integr. Plant. Biol. 66: 143–159. 37975264 10.1111/jipb.13586

[jipb70351-bib-0064] Liu, J. , Shi, Y. , and Yang, S. (2018a). Insights into the regulation of C‐repeat binding factors in plant cold signaling. J. Integr. Plant. Biol. 60: 780–795. 29667328 10.1111/jipb.12657

[jipb70351-bib-0065] Liu, L. , Hao, J. , Huang, K. , Duan, P. , Zhang, B. , Chi, Z. , Yao, X. , and Li, Y. (2025a). Redox regulation of G protein oligomerization and signaling by the glutaredoxin WG1 controls grain size in rice. EMBO J. 44: 3742–3763. 40389777 10.1038/s44318-025-00462-9 PMC12216599

[jipb70351-bib-0066] Liu, Q. , Han, R. , Wu, K. , Zhang, J. , Ye, Y. , Wang, S. , Chen, J. , Pan, Y. , Li, Q. , Xu, X. , et al. (2018b). G‐protein βγ subunits determine grain size through interaction with MADS‐domain transcription factors in rice. Nat. Commun. 9: 852. 29487282 10.1038/s41467-018-03047-9 PMC5829230

[jipb70351-bib-0067] Liu, Y. , Chen, N. , Fan, X. , Xia, X. , Yao, Y. , Huang, W. , Sun, J. , Huang, L. , Wang, L. , Yu, H. , et al. (2025b). *SP3* and *DEP1* orchestrate panicle architecture by jointly regulating *APO2* expression in rice. Adv. Sci. 12: e08230. 10.1002/advs.202508230 PMC12667520 40899627

[jipb70351-bib-0068] Luo, W. , Huan, Q. , Xu, Y. , Qian, W. , Chong, K. , and Zhang, J. (2021). Integrated global analysis reveals a vitamin E‐vitamin K1 sub‐network, downstream of COLD1, underlying rice chilling tolerance divergence. Cell Rep. 36: 109397. 34289369 10.1016/j.celrep.2021.109397

[jipb70351-bib-0069] Ma, A. , Zhang, Y. , Wang, Y. , Ma, H. , Chen, H. , Qi, Y. , Zhang, M. , Zhong, Z. , Cheng, J. , Qi, J. , et al. (2025a). ZmCRK5A kinase enhances drought tolerance in maize via phosphorylation‐dependent inhibition of ZmSMH4. J. Integr. Plant. Biol. 68: 23–38. 41131956 10.1111/jipb.70054

[jipb70351-bib-0070] Ma, M. , Wang, W. , Fei, Y. , Cheng, H.‐Y. , Song, B. , Zhou, Z. , Zhao, Y. , Zhang, X. , Li, L. , Chen, S. , et al. (2022). A surface‐receptor‐coupled G protein regulates plant immunity through nuclear protein kinases. Cell Host Microbe 30: 1602–1614. 36240763 10.1016/j.chom.2022.09.012

[jipb70351-bib-0071] Ma, M. , Zhou, J.‐M. , and Liang, X. (2024). Phosphorylation‐dependent regulation of plant heterotrimeric G proteins: From activation to downstream signaling. Sci. Bull. 69: 2967–2970. 10.1016/j.scib.2024.04.067 38760249

[jipb70351-bib-0072] Ma, X. , Wang, W. , Zhang, J. , Jiang, Z. , Xu, C. , Zhu, W. , Shi, B. , Yang, W. , Su, H. , Wang, X. , et al. (2025b). NRT1.1B acts as an abscisic acid receptor in integrating compound environmental cues for plants. Cell 188: 5231–5248. 40795855 10.1016/j.cell.2025.07.027

[jipb70351-bib-0073] Ma, Y. , Chen, M. , Xu, D. , Fang, G. , Wang, E. , Gao, S. , Xu, Z. , Li, L. , Zhang, X. , Min, D. , et al. (2015a). G‐protein β subunit AGB1 positively regulates salt stress tolerance in Arabidopsis. J. Integr. Agric. 14: 314–325.

[jipb70351-bib-0074] Ma, Y. , Dai, X. , Xu, Y. , Luo, W. , Zheng, X. , Zeng, D. , Pan, Y. , Lin, X. , Liu, H. , Zhang, D. , et al. (2015b). *COLD1* confers chilling tolerance in rice. Cell 160: 1209–1221. 25728666 10.1016/j.cell.2015.01.046

[jipb70351-bib-0075] Mao, H. , Sun, S. , Yao, J. , Wang, C. , Yu, S. , Xu, C. , Li, X. , and Zhang, Q. (2010). Linking differential domain functions of the GS3 protein to natural variation of grain size in rice. Proc. Natl. Acad. Sci. U.S.A. 107: 19579–19584. 20974950 10.1073/pnas.1014419107 PMC2984220

[jipb70351-bib-0076] McFarlane, H.E. , Mutwil‐Anderwald, D. , Verbančič, J. , Picard, K.L. , Gookin, T.E. , Froehlich, A. , Chakravorty, D. , Trindade, L.M. , Alonso, J.M. , Assmann, S.M. , et al. (2021). A G protein‐coupled receptor‐like module regulates cellulose synthase secretion from the endomembrane system in Arabidopsis. Dev. Cell 56: 1484–1497. 33878345 10.1016/j.devcel.2021.03.031 PMC8754689

[jipb70351-bib-0077] Miao, J. , Ran, J. , Xu, M. , Bo, L. , Wang, P. , Liang, G. , and Zhou, Y. (2024). Overexpression of *RGG2*, a Heterotrimeric G protein γ subunit‐encoding gene, improves drought tolerance in rice. Chin. J. Rice Sci. 38: 246.

[jipb70351-bib-0078] Miao, J. , Yang, Z. , Zhang, D. , Wang, Y. , Xu, M. , Zhou, L. , Wang, J. , Wu, S. , Yao, Y. , Du, X. , et al. (2019). Mutation of *RGG2*, which encodes a type B heterotrimeric G protein γ subunit, increases grain size and yield production in rice. Plant Biotechnol. J. 17: 650–664. 30160362 10.1111/pbi.13005 PMC6381795

[jipb70351-bib-0079] Milligan, G. , and Kostenis, E. (2006). Heterotrimeric G‐proteins: A short history. Br. J. Pharmacol. 147: S46–S55. 16402120 10.1038/sj.bjp.0706405 PMC1760735

[jipb70351-bib-0080] Nakashima, K. , Ito, Y. , and Yamaguchi‐Shinozaki, K. (2009). Transcriptional regulatory networks in response to abiotic stresses in Arabidopsis and grasses. Plant Physiol. 149: 88–95. 19126699 10.1104/pp.108.129791 PMC2613698

[jipb70351-bib-0081] Nilson, S.E. , and Assmann, S.M. (2010). The α‐subunit of the Arabidopsis heterotrimeric G protein, GPA1, is a regulator of transpiration efficiency. Plant Physiol. 152: 2067–2077. 20200073 10.1104/pp.109.148262 PMC2850002

[jipb70351-bib-0082] Ninh, T.T. , Gao, W. , Trusov, Y. , Zhao, J.‐R. , Long, L. , Song, C.‐P. , and Botella, J.R. (2021). Tomato and cotton G protein beta subunit mutants display constitutive autoimmune responses. Plant Direct 5: e359. 34765865 10.1002/pld3.359 PMC8573408

[jipb70351-bib-0083] Northup, J.K. , Sternweis, P.C. , Smigel, M.D. , Schleifer, L.S. , Ross, E.M. , and Gilman, A.G. (1980). Purification of the regulatory component of adenylate cyclase. Proc. Natl. Acad. Sci. U.S.A. 77: 6516–6520. 6935665 10.1073/pnas.77.11.6516 PMC350316

[jipb70351-bib-0084] Oki, K. , Fujisawa, Y. , Kato, H. , and Iwasaki, Y. (2005). Study of the constitutively active form of the alpha subunit of rice heterotrimeric G proteins. Plant Cell Physiol. 46: 381–386. 15695461 10.1093/pcp/pci036

[jipb70351-bib-0085] Peng, P. , Gao, Y. , Li, Z. , Yu, Y. , Qin, H. , Guo, Y. , Huang, R. , and Wang, J. (2019). Proteomic analysis of a rice mutant *sd58* possessing a novel *d1* allele of heterotrimeric G protein alpha subunit (RGA1) in salt stress with a focus on ROS scavenging. Int. J. Mol. Sci. 20: 167. 30621186 10.3390/ijms20010167 PMC6337198

[jipb70351-bib-0086] Perrotta, R.M. , Vinke, S. , Ferreira, R. , Moret, M. , Mahas, A. , Chiappino‐Pepe, A. , Riedmayr, L.M. , Mehra, A.‐T. , Lehmann, L.S. , Church, G.M. (2025). Engineered base editors with reduced bystander editing through directed evolution. Nat. Biotechnol. 2025 10.1038/s41587-025-02937-w 41413244

[jipb70351-bib-0087] Pillai, A. , Idris, A. , Philomin, A. , Weidle, C. , Skotheim, R. , Leung, P.J.Y. , Broerman, A. , Demakis, C. , Borst, A.J. , Praetorius, F. , et al. (2024). *De novo* design of allosterically switchable protein assemblies. Nature 632: 911–920. 39143214 10.1038/s41586-024-07813-2 PMC11338832

[jipb70351-bib-0088] Rankenberg, T. , van Veen, H. , Sedaghatmehr, M. , Liao, C.‐Y. , Devaiah, M.B. , Stouten, E.A. , Balazadeh, S. , and Sasidharan, R. (2024). Differential leaf flooding resilience in *Arabidopsis thaliana* is controlled by ethylene signaling‐activated and age‐dependent phosphorylation of ORESARA1. Plant Commun. 5: 100848. 38379284 10.1016/j.xplc.2024.100848 PMC11211547

[jipb70351-bib-0089] Ren, D. , Ding, C. , and Qian, Q. (2023). Molecular bases of rice grain size and quality for optimized productivity. Sci. Bull. 68: 314–350. 10.1016/j.scib.2023.01.026 36710151

[jipb70351-bib-0090] Rico, J.A.F. , and Höcker, B. (2013). Design of chimeric proteins by combination of subdomain‐sized fragments. Methods Enzymol. 523: 389–405. 23422440 10.1016/B978-0-12-394292-0.00018-7

[jipb70351-bib-0091] Roy Choudhury, S. , and Pandey, S. (2016). Interaction of heterotrimeric G‐protein components with receptor‐like kinases in plants: An alternative to the established signaling paradigm? Mol. Plant 9: 1093–1095. 27250573 10.1016/j.molp.2016.05.012

[jipb70351-bib-0092] Sakamoto, T. , and Matsuoka, M. (2008). Identifying and exploiting grain yield genes in rice. Curr. Opin. Plant Biol. 11: 209–214. 18343712 10.1016/j.pbi.2008.01.009

[jipb70351-bib-0093] Savary, S. , Willocquet, L. , Pethybridge, S.J. , Esker, P. , McRoberts, N. , and Nelson, A. (2019). The global burden of pathogens and pests on major food crops. Nat. Ecol. Evol. 3: 430–439. 30718852 10.1038/s41559-018-0793-y

[jipb70351-bib-0094] Seo, E. , Lee, H. , Jeon, J. , Park, H. , Kim, J. , Noh, Y.‐S. , and Lee, I. (2009). Crosstalk between cold response and flowering in Arabidopsis is mediated through the flowering‐time gene *SOC1* and its upstream negative regulator FLC. Plant Cell 21: 3185–3197. 19825833 10.1105/tpc.108.063883 PMC2782271

[jipb70351-bib-0095] Shen, Y. , Yang, G. , Miao, X. , and Shi, Z. (2023). OsmiR159 modulate BPH resistance through regulating G‐protein γ subunit *GS3* gene in rice. Rice 16: 30. 37402009 10.1186/s12284-023-00646-z PMC10319700

[jipb70351-bib-0096] Shi, Y. , Xue, R. , Zheng, Q. , Guo, Z. , Wang, C. , Zheng, L. , Li, Y. , Yang, J. , Jin, W. , Tang, J. , et al. (2025). CT2 is involved in yield‐related traits and cell proliferation of maize. J. Genet. 104: 17. 40667626

[jipb70351-bib-0097] Sinha, S. , Tam, B. , and Wang, S.M. (2022). Applications of molecular dynamics simulation in protein study. Membranes 12: 844. 36135863 10.3390/membranes12090844 PMC9505860

[jipb70351-bib-0098] Subramaniam, G. , Trusov, Y. , Lopez‐Encina, C. , Hayashi, S. , Batley, J. , and Botella, J.R. (2016). Type B heterotrimeric G protein γ‐subunit regulates auxin and ABA signaling in tomato. Plant Physiol. 170: 1117–1134. 26668332 10.1104/pp.15.01675 PMC4734580

[jipb70351-bib-0099] Sun, H. , Qian, Q. , Wu, K. , Luo, J. , Wang, S. , Zhang, C. , Ma, Y. , Liu, Q. , Huang, X. , Yuan, Q. , et al. (2014). Heterotrimeric G proteins regulate nitrogen‐use efficiency in rice. Nat. Genet. 46: 652–656. 24777451 10.1038/ng.2958

[jipb70351-bib-0100] Sun, L. , Zhang, C. , Dong, J. , Huang, W. , Jin, F. , Li, C. , Zhou, W. , Wu, W. , and Li, X. (2026). Genome‐wide identification of maize G protein genes and regulatory roles of the *ZmGG1* subfamily in saline‐alkali stress response. BMC Plant Biol. 26: 693. 41803690 10.1186/s12870-026-08509-7 PMC13085601

[jipb70351-bib-0101] Sun, S. , Cheng, J. , Zhang, Y. , Wang, Y. , Wang, L. , Wang, T. , Wang, Z. , Li, X. , Zhou, Y. , Li, X. , et al. (2025). Novel repetitive elements in plant‐specific tails of Gγ proteins as the functional unit in G‐protein signaling in crops. Plant Cell 37: koaf052. 40088466 10.1093/plcell/koaf052 PMC12070394

[jipb70351-bib-0102] Sun, S. , Wang, L. , Mao, H. , Shao, L. , Li, X. , Xiao, J. , Ouyang, Y. , and Zhang, Q. (2018). A G‐protein pathway determines grain size in rice. Nat. Commun. 9: 851. 29487318 10.1038/s41467-018-03141-y PMC5829277

[jipb70351-bib-0103] Sun, W. , Zhang, H. , Yang, S. , Liu, L. , Xie, P. , Li, J. , Zhu, Y. , Ouyang, Y. , Xie, Q. , Zhang, H. , et al. (2023). Genetic modification of Gγ subunit AT1 enhances salt‐alkali tolerance in main graminaceous crops. Natl. Sci. Rev. 10: nwad075. 37181090 10.1093/nsr/nwad075 PMC10171625

[jipb70351-bib-0104] Swain, D.M. , Sahoo, R.K. , Srivastava, V.K. , Tripathy, B.C. , Tuteja, R. , and Tuteja, N. (2017). Function of heterotrimeric G‐protein γ subunit RGG1 in providing salinity stress tolerance in rice by elevating detoxification of ROS. Planta 245: 367–383. 27785615 10.1007/s00425-016-2614-3

[jipb70351-bib-0105] Tao, Y. , Miao, J. , Wang, J. , Li, W. , Xu, Y. , Wang, F. , Jiang, Y. , Chen, Z. , Fan, F. , Xu, M. , et al. (2020). RGG1, involved in the cytokinin regulatory pathway, controls grain size in rice. Rice 13: 76. 33169285 10.1186/s12284-020-00436-x PMC7652983

[jipb70351-bib-0106] Tao, Y. , Trusov, Y. , Zhao, X. , Wang, X. , Cruickshank, A.W. , Hunt, C. , van Oosterom, E.J. , Hathorn, A. , Liu, G. , Godwin, I.D. , et al. (2021). Manipulating assimilate availability provides insight into the genes controlling grain size in sorghum. Plant J. 108: 231–243. 34309934 10.1111/tpj.15437

[jipb70351-bib-0107] Temple, B.R.S. , and Jones, A.M. (2007). The plant heterotrimeric G‐protein complex. Annu. Rev. Plant Biol. 58: 249–266. 17201690 10.1146/annurev.arplant.58.032806.103827

[jipb70351-bib-0108] Tuninetti, M. , and Davis, K.F. (2026). Addressing global hotspots of drought‐related crop production losses. Nat. Commun. 17: 6605. 42156736 10.1038/s41467-026-72715-y PMC13381596

[jipb70351-bib-0109] Ueguchi‐Tanaka, M. , Fujisawa, Y. , Kobayashi, M. , Ashikari, M. , Iwasaki, Y. , Kitano, H. , and Matsuoka, M. (2000). Rice dwarf mutant *d1*, which is defective in the α subunit of the heterotrimeric G protein, affects gibberellin signal transduction. Proc. Natl. Acad. Sci. U.S.A. 97: 11638–11643. 11027362 10.1073/pnas.97.21.11638 PMC17253

[jipb70351-bib-0110] Ullah, H. , Chen, J.‐G. , Wang, S. , and Jones, A.M. (2002). Role of a heterotrimeric G protein in regulation of Arabidopsis seed germination. Plant Physiol. 129: 897–907. 12068128 10.1104/pp.005017 PMC161710

[jipb70351-bib-0111] Urano, D. , Colaneri, A. , and Jones, A.M. (2014). Gα modulates salt‐induced cellular senescence and cell division in rice and maize. J. Exp. Bot. 65: 6553–6561. 25227951 10.1093/jxb/eru372 PMC4246186

[jipb70351-bib-0112] Urano, D. , Jones, J.C. , Wang, H. , Matthews, M. , Bradford, W. , Bennetzen, J.L. , and Jones, A.M. (2012). G protein activation without a GEF in the plant kingdom. PLoS Genet. 8: e1002756. 22761582 10.1371/journal.pgen.1002756 PMC3386157

[jipb70351-bib-0113] Wang, C. , Tang, S. , Zhan, Q. , Hou, Q. , Zhao, Y. , Zhao, Q. , Feng, Q. , Zhou, C. , Lyu, D. , Cui, L. , et al. (2019b). Dissecting a heterotic gene through GradedPool‐Seq mapping informs a rice‐improvement strategy. Nat. Commun. 10: 2982. 31278256 10.1038/s41467-019-11017-y PMC6611799

[jipb70351-bib-0114] Wang, J. , Luo, Q. , Deng, J. , Liang, X. , Li, Y. , Wang, A. , Lin, T. , Liu, H. , Zhang, X. , Liu, Z. , et al. (2025b). The G‐protein β subunit *SlGB1* regulates tyramine‐derived phenolamide metabolism for shoot apex growth and development in tomato. Plant Cell 37: koaf070. 40152502 10.1093/plcell/koaf070 PMC11983129

[jipb70351-bib-0115] Wang, S. , Li, S. , Liu, Q. , Wu, K. , Zhang, J. , Wang, S. , Wang, Y. , Chen, X. , Zhang, Y. , Gao, C. , et al. (2015). The OsSPL16‐GW7 regulatory module determines grain shape and simultaneously improves rice yield and grain quality. Nat. Genet. 47: 949–954. 26147620 10.1038/ng.3352

[jipb70351-bib-0116] Wang, S. , Wu, K. , Yuan, Q. , Liu, X. , Liu, Z. , Lin, X. , Zeng, R. , Zhu, H. , Dong, G. , Qian, Q. , et al. (2012). Control of grain size, shape and quality by OsSPL16 in rice. Nat. Genet. 44: 950–954. 22729225 10.1038/ng.2327

[jipb70351-bib-0117] Wang, X.‐Q. , Ullah, H. , Jones, A.M. , and Assmann, S.M. (2001). G protein regulation of ion channels and abscisic acid signaling in Arabidopsis guard cells. Science 292: 2070–2072. 11408655 10.1126/science.1059046

[jipb70351-bib-0118] Wang, Y. , Lv, Y. , Wen, Y. , Wang, J. , Hu, P. , Wu, K. , Chai, B. , Gan, S. , Liu, J. , Wu, Y. , et al. (2025a). GS2 cooperates with IPA1 to control panicle architecture. New Phytol. 245: 2726–2743. 39887382 10.1111/nph.20412 PMC11840411

[jipb70351-bib-0119] Wang, Y. , Lv, Y. , Yu, H. , Hu, P. , Wen, Y. , Wang, J. , Tan, Y. , Wu, H. , Zhu, L. , Wu, K. , et al. (2024a). *GR5* acts in the G protein pathway to regulate grain size in rice. Plant Commun. 5: 100673. 37596786 10.1016/j.xplc.2023.100673 PMC10811372

[jipb70351-bib-0120] Wang, Y. , Wang, Y. , and Deng, D. (2019a). Multifaceted plant G protein: Interaction network, agronomic potential, and beyond. Planta 249: 1259–1266. 30790051 10.1007/s00425-019-03112-7

[jipb70351-bib-0121] Wang, Y. , Zhang, H. , Wang, P. , Zhong, H. , Liu, W. , Zhang, S. , Xiong, L. , Wu, Y. , and Xia, Y. (2023). Arabidopsis EXTRA‐LARGE G PROTEIN 1 (XLG1) functions together with XLG2 and XLG3 in PAMP‐triggered MAPK activation and immunity. J. Integr. Plant Biol. 65: 825–837. 36250681 10.1111/jipb.13391

[jipb70351-bib-0122] Wang, Z. , Zhang, X. , Liu, C. , Duncan, S. , Hang, R. , Sun, J. , Luo, L. , Ding, Y. , and Cao, X. (2024b). AtPRMT3‐RPS2B promotes ribosome biogenesis and coordinates growth and cold adaptation trade‐off. Nat. Commun. 15: 8693. 39375381 10.1038/s41467-024-52945-8 PMC11488217

[jipb70351-bib-0123] Wang, Y. , Zhu, L. , Yang, Y. , Li, X. , Zhang, X. , and Fang, X. (2026). Cellular water‐potential sensing through biomolecular condensation. Nature 655: 1320–1329. 42203875 10.1038/s41586-026-10591-8 PMC13421324

[jipb70351-bib-0124] Wu, Q. , Regan, M. , Furukawa, H. , and Jackson, D. (2018). Role of heterotrimeric Gα proteins in maize development and enhancement of agronomic traits. PLoS Genet. 14: e1007374. 29708966 10.1371/journal.pgen.1007374 PMC5945058

[jipb70351-bib-0125] Wu, Q. , Xu, F. , Liu, L. , Char, S.N. , Ding, Y. , Je, B.I. , Schmelz, E. , Yang, B. , and Jackson, D. (2020). The maize heterotrimeric G protein β subunit controls shoot meristem development and immune responses. Proc. Natl. Acad. Sci. U. S. A. 117: 1799–1805. 31852823 10.1073/pnas.1917577116 PMC6983446

[jipb70351-bib-0126] Xiao, W. , Wang, H. , Wang, M. , and Wang, J. (2024). Mechanisms of plant response to saline‐alkali stress: A Review. Chin. Agric. Sci. Bull. 40: 78.

[jipb70351-bib-0127] Xie, P. , Liu, F. , and Xie, Q. (2024). Manipulating hormones to mitigate trade‐offs in crops. Plant, Cell Envir 47: 4903–4907. 10.1111/pce.15076 39101664

[jipb70351-bib-0128] Xie, P. , Tang, S. , Chen, C. , Zhang, H. , Yu, F. , Li, C. , Wei, H. , Sui, Y. , Wu, C. , Diao, X. , et al. (2022). Natural variation in Glume Coverage 1 causes naked grains in sorghum. Nat. Commun. 13: 1068. 35217660 10.1038/s41467-022-28680-3 PMC8881591

[jipb70351-bib-0129] Xie, P. , Wu, Y. , and Xie, Q. (2023). Evolution of cereal floral architecture and threshability. Trends Plant Sci. 28: 1438–1450. 37673701 10.1016/j.tplants.2023.08.003

[jipb70351-bib-0130] Xing, Y. , and Zhang, Q. (2010). Genetic and molecular bases of rice yield. Annu. Rev. Plant Biol. 61: 421–442. 20192739 10.1146/annurev-arplant-042809-112209

[jipb70351-bib-0131] Xiong, X.X. , Liu, Y. , Zhang, L.‐L. , Li, X.J. , Zhao, Y. , Zheng, Y. , Yang, Q.H. , Yang, Y. , Min, D.H. , and Zhang, X.H. (2023). G‐protein β‐Subunit gene *TaGB1‐B* enhances drought and salt resistance in wheat. Int. J. Mol. Sci. 24: 7337. 37108500 10.3390/ijms24087337 PMC10138664

[jipb70351-bib-0132] Xu, D. , Chen, M. , Ma, Y. , Xu, Z. , Li, L. , Chen, Y. , and Ma, Y. (2015). a G‐protein β subunit, AGB1, negatively regulates the ABA response and drought tolerance by down‐regulating *AtMPK6*‐related pathway in Arabidopsis. PLoS ONE 10: e0116385. 25635681 10.1371/journal.pone.0116385 PMC4312036

[jipb70351-bib-0133] Xu, H. , Zhao, M. , Zhang, Q. , Xu, Z. , and Xu, Q. (2016). The *DENSE AND ERECT PANICLE 1* (*DEP1*) gene offering the potential in the breeding of high‐yielding rice. Breed. Sci. 66: 659–667. 28163581 10.1270/jsbbs.16120 PMC5282764

[jipb70351-bib-0134] Xu, P. , Lian, H. , Xu, F. , Zhang, T. , Wang, S. , Wang, W. , Du, S. , Huang, J. , and Yang, H.Q. (2019). Phytochrome B and AGB1 coordinately regulate photomorphogenesis by antagonistically modulating PIF3 stability in Arabidopsis. Mol. Plant 12: 229–247. 30576873 10.1016/j.molp.2018.12.003

[jipb70351-bib-0135] Xue, J. , Gong, B.Q. , Yao, X. , Huang, X. , and Li, J.F. (2020). BAK1‐mediated phosphorylation of canonical G protein alpha during flagellin signaling in Arabidopsis. J. Integr. Plant Biol 62: 690–701. 31087771 10.1111/jipb.12824

[jipb70351-bib-0136] Yadav, D.K. , Islam, S.M.S. , and Tuteja, N. (2012). Rice heterotrimeric G‐protein gamma subunits (RGG1 and RGG2) are differentially regulated under abiotic stress. Plant Signal. Behav. 7: 733–740. 22751322 10.4161/psb.20356 PMC3583952

[jipb70351-bib-0137] Yadav, P. , Khatri, N. , Gupta, R. , and Mudgil, Y. (2024). Proteomic profiling of Arabidopsis G‐protein β subunit AGB1 mutant under salt stress. Physiol. Mol. Biol. Plants 30: 571–586. 38737318 10.1007/s12298-024-01448-3 PMC11087450

[jipb70351-bib-0138] Yang, W. , Wu, K. , Wang, B. , Liu, H. , Guo, S. , Guo, X. , Luo, W. , Sun, S. , Ouyang, Y. , Fu, X. , et al. (2021). The RING E3 ligase CLG1 targets GS3 for degradation via the endosome pathway to determine grain size in rice. Mol. Plant 14: 1699–1713. 34216830 10.1016/j.molp.2021.06.027

[jipb70351-bib-0139] Yang, X. , Lu, J. , Shi, W.‐J. , Chen, Y.H. , Yu, J.W. , Chen, S.H. , Zhao, D.S. , Huang, L.C. , Fan, X.L. , Zhang, C.Q. , et al. (2024). RGA1 regulates grain size, rice quality and seed germination in the small and round grain mutant srg5. BMC Plant Biol. 24: 167. 38438916 10.1186/s12870-024-04864-5 PMC10910726

[jipb70351-bib-0140] Yaseen, M. , Tariq, N. , Kanwal, R. , Farooq, A. , Wang, H. , and Yuan, H. (2025). Rice grain size: Current regulatory mechanisms and future perspectives. J. Plant Res. 138: 403–417. 40056359 10.1007/s10265-025-01626-8

[jipb70351-bib-0141] Yu, J. , Miao, J. , Zhang, Z. , Xiong, H. , Zhu, X. , Sun, X. , Pan, Y. , Liang, Y. , Zhang, Q. , Abdul Rehman, R.M. , et al. (2018a). Alternative splicing of OsLG3b controls grain length and yield in japonica rice. Plant Biotechnol. J. 16: 1667–1678. 29479793 10.1111/pbi.12903 PMC6097128

[jipb70351-bib-0142] Yu, Q. , Wang, B. , Li, N. , Tang, Y. , Yang, S. , Yang, T. , Xu, J. , Guo, C. , Yan, P. , Wang, Q. , et al. (2017). CRISPR/Cas9‐induced targeted mutagenesis and gene replacement to generate long‐shelf life tomato lines. Sci. Rep. 7: 11874. 28928381 10.1038/s41598-017-12262-1 PMC5605656

[jipb70351-bib-0143] Yu, Y. , and Assmann, S.M. (2018). Inter‐relationships between the heterotrimeric Gβ subunit AGB1, the receptor‐like kinase FERONIA, and RALF1 in salinity response. Plant Cell Environ. 41: 2475–2489. 29907954 10.1111/pce.13370 PMC6150805

[jipb70351-bib-0144] Yu, Y. , Chakravorty, D. , and Assmann, S.M. (2018b). The G protein β‐subunit, AGB1, interacts with FERONIA in RALF1‐regulated stomatal movement. Plant Physiol. 176: 2426–2440. 29301953 10.1104/pp.17.01277 PMC5841690

[jipb70351-bib-0145] Yuan, G.L. , Li, H.J. , and Yang, W.C. (2017). The integration of Gβ and MAPK signaling cascade in zygote development. Sci. Rep. 7: 8732. 28821747 10.1038/s41598-017-08230-4 PMC5562876

[jipb70351-bib-0146] Yuan, H. , Cheng, M. , Wang, R. , Wang, Z. , Fan, F. , Wang, W. , Si, F. , Gao, F. , and Li, S. (2024). miR396b/GRF6 module contributes to salt tolerance in rice. Plant Biotechnol. J. 22: 2079–2092. 38454780 10.1111/pbi.14326 PMC11258987

[jipb70351-bib-0147] Zeng, D. , Tian, Z. , Rao, Y. , Dong, G. , Yang, Y. , Huang, L. , Leng, Y. , Xu, J. , Sun, C. , Zhang, G. , et al. (2017). Rational design of high‐yield and superior‐quality rice. Nat. Plants 3: 17031. 28319055 10.1038/nplants.2017.31

[jipb70351-bib-0148] Zhang, D. , Zhang, M. , and Liang, J. (2021). RGB1 regulates grain development and starch accumulation through its effect on OsYUC11‐mediated auxin biosynthesis in rice endosperm cells. Front. Plant Sci. 12: 585174. 33868323 10.3389/fpls.2021.585174 PMC8045708

[jipb70351-bib-0149] Zhang, D.P. , Zhou, Y. , Yin, J.F. , Yan, X.J. , Lin, S. , Xu, W.F. , Baluška, F. , Wang, Y.P. , Xia, Y.J. , Liang, G. , et al. (2015). Rice G‐protein subunits qPE9‐1 and RGB1 play distinct roles in abscisic acid responses and drought adaptation. J. Exp. Bot. 66: 6371–6384. 26175353 10.1093/jxb/erv350

[jipb70351-bib-0150] Zhang, H. , Yu, F. , Xie, P. , Sun, S. , Qiao, X. , Tang, S. , Chen, C. , Yang, S. , Mei, C. , Yang, D. , et al. (2023). A Gγ protein regulates alkaline sensitivity in crops. Science 379: eade8416. 36952416 10.1126/science.ade8416

[jipb70351-bib-0151] Zhang, H. , Zhao, Y. , and Zhu, J.K. (2020). Thriving under stress: How plants balance growth and the stress response. Dev. Cell 55: 529–543. 33290694 10.1016/j.devcel.2020.10.012

[jipb70351-bib-0152] Zhang, J. , Lin, Q. , Wang, X. , Shao, J. , Ren, Y. , Liu, X. , Feng, M. , Li, S. , Sun, Q. , Luo, S. , et al. (2025b). The DENSE AND ERECT PANICLE1‐GRAIN NUMBER ASSOCIATED module enhances rice yield by repressing *CYTOKININ OXIDASE 2* expression. Plant Cell 37: koae309. 10.1093/plcell/koae309 PMC11663551 39660553

[jipb70351-bib-0153] Zhang, Y. , Yang, Z. , Bai, W. , Huang, S. , Chen, Y. , and Sun, J. (2025a). Heterotrimeric G protein interacts with MLO1 to regulate a trade‐off between disease resistance and growth in wheat. Physiol. Plant. 177: e70100. 39903056 10.1111/ppl.70100

[jipb70351-bib-0154] Zhao, H. , Chen, Y. , Li, W. , Ding, Y. , and Wang, Y. (2025a). Plasma membrane maize Gγ protein MGG4 positively regulates seed size mainly through influencing kernel width. Plant Cell Rep. 44: 210. 40928658 10.1007/s00299-025-03594-8

[jipb70351-bib-0155] Zhao, J. , and Wang, X. (2004). Arabidopsis phospholipase Dα1 interacts with the heterotrimeric G‐protein α‐subunit through a motif analogous to the DRY motif in G‐protein‐coupled receptors. J. Biol. Chem. 279: 1794–1800. 14594812 10.1074/jbc.M309529200

[jipb70351-bib-0156] Zhao, Y. , Liang, Y. , Ni, Z. , Sun, Q. , Zong, Y. , and Wang, Y. (2025b). Advances and prospects of large DNA fragment editing in plants. Nat. Plants 11: 2461–2475. 41286485 10.1038/s41477-025-02160-0

[jipb70351-bib-0157] Zhao, Y. , Shi, Y. , Jiang, G. , Wu, Y. , Ma, M. , Zhang, X. , Liang, X. , and Zhou, J.M. (2022). Rice extra‐large G proteins play pivotal roles in controlling disease resistance and yield‐related traits. New Phytol. 234: 607–617. 35090194 10.1111/nph.17997

[jipb70351-bib-0158] Zhou, F. , Guo, Z. , Ling, Y. , and Cheng, Y. (2025b). G proteins regulate rice grain size by modulating the Hippo signaling pathway. Plant Cell 24: 2025. 10.1093/plcell/koaf288 41442556

[jipb70351-bib-0159] Zhou, T. , Chen, H. , Jiang, X. , Zhu, H. , Lin, Q. , Jung, K.‐H. , Zhu, X. , and Xuan, Y.H. (2025a). TCP19 regulates nitrogen‐dependent sheath blight susceptibility by modulating nitrogen uptake and signaling in rice. Plant Biotechnol. J. 23: 4140–4157. 40567041 10.1111/pbi.70224 PMC12392934

[jipb70351-bib-0160] Zou, G. , Zhai, G. , Yan, S. , Li, S. , Zhou, L. , Ding, Y. , Liu, H. , Zhang, Z. , Zou, J. , Zhang, L. , et al. (2020). Sorghum *qTGW1a* encodes a G‐protein subunit and acts as a negative regulator of grain size. J. Exp. Bot. 71: 5389–5401. 32497208 10.1093/jxb/eraa277

